# Transcriptome and 16S rRNA Amplicon Sequencing Analysis of Nutrition Metabolism in Silver Pomfret at Varying Flow Rates

**DOI:** 10.3390/ani16121818

**Published:** 2026-06-12

**Authors:** Jiabao Hu, Yuanbo Li, Youyi Zhang, Rongyue Zheng, Xiaojun Yan, Man Zhang, Yajun Wang, Lingling Jia

**Affiliations:** 1 College of Marine Sciences, College of Food Science and Engineering, Ningbo University, Ningbo 315832, China; hujiabao10@163.com (J.H.); liyuanbo0717@163.com (Y.L.); zyy970805@163.com (Y.Z.); yanxiaojun@nbu.edu.cn (X.Y.); zhangman@nbu.edu.cn (M.Z.); wangyajun@nbu.edu.cn (Y.W.); 2 School of Civil & Environmental Engineering and Geography Science, Ningbo University, Ningbo 315832, China; zhengrongyue@nbu.edu.cn; 3 Key Laboratory of Applied Marine Biotechnology, Ningbo University, Ministry of Education, Ningbo 315832, China; 4 Key Laboratory of Marine Biotechnology of Zhejiang Province, Ningbo University, Ningbo 315832, China; 5 XiangShan (NingBo University) Aquatic Seed Industry Innovation Research Institute, Ningbo 315832, China

**Keywords:** recirculating aquaculture, transcriptome, 16S rRNA sequencing, *Pampus argenteus*

## Abstract

Silver pomfret (*Pampus argenteus*), a highly valued marine fish, faces challenges in aquaculture due to its sensitivity to environmental conditions. Recirculating aquaculture system (RAS) is likely to become a primary indoor cultivation method for silver pomfret in the future, so studying hydrodynamic characteristics at varying flow rates in silver pomfret RAS is crucial and has far-reaching implications for both aquaculture practices and economic returns. This study investigated the effects of water flow rates (low: 400 L/h, moderate: 600 L/h, high: 800 L/h) on the growth performance, nutritional metabolism, and gut microbiota of silver pomfret over an 8-week period. Transcriptome and 16S rRNA sequencing revealed that the moderate-flow-rate group exhibited superior growth performance, with enhanced expression of genes related to protein synthesis (HYOU1, PDIA6, ITGA11) and redox regulation (GLUL, DUSP1, GST). Additionally, the moderate flow rate promoted gut microbial diversity and stability, with higher abundances of fermentation- and chemoheterotrophy-related functions, suggesting improved nutrient metabolism. In contrast, high flow rates induced anaerobic metabolism, leading to lactate accumulation and reduced growth. These findings demonstrate that a moderate flow rate of 600 L/h optimizes silver pomfret aquaculture by enhancing growth, metabolic efficiency, and microbial health, providing a foundation for sustainable large-scale farming practices.

## Simple Summary

This study found that a moderate water flow rate (600 L/h) was best for growing silver pomfret in a recirculating aquaculture system. Fish in this group showed better growth, healthier metabolism, and more stable gut microbes than those in low- or high-flow conditions. These results suggest that using a moderate flow rate can improve fish health and support more sustainable silver pomfret farming.

## 1. Introduction

Silver pomfret (*Pampus argenteus*), as a highly economic marine fish species, is widely distributed in the coastal seas of Indo-West Pacifc: Persian Gulf to Indonesia, West and Southwest of Korea and almost the entire coastal area of China, north to Hokkaido, Japan [1]. The fish meat is very delicious and has extremely high nutritional value, and it is very convenient for older people and children to eat due to the lack of intermuscular bone and soft bones. Thus, this fish becomes one of the most preferred edible fishes in many Asian countries. Successful taming and breeding of young fish have been achieved since 2000 by our research team. By 2018, more than ten thousand adult fish were cultured, and nearly a million young fish were bred [2]. During the culturing process, parasites (*Amyloodinium ocellatum*) [3], bacterium [4,5], and virus (*iridovirus*) diseases [6] were identified as the main threats to this fish in high-temperature seasons, leading to an alarmingly high mortality rate. By implementing an indoor flowing-water culture method to control water quality, we successfully reduced the outbreak rate of these diseases. As a result, we significantly improved the survival rate of this fish to 80% in 2023. Thus, flow rate plays a key role in silver pomfret survival rate in this culture mode. At present, there are limited studies about the effect of flow rate on silver pomfret, which is worthy of further research.

The flow of water can stimulate various sensory responses in fish, eliciting corresponding reaction mechanisms [7], and the swimming behavior of fish, influenced by their tendency to follow currents, is a crucial aspect of their life activities. Studying the swimming behaviors of fish at varying flow rates holds significant importance for understanding their growth, predation, reproduction, fishing practices, and the construction of fish passages. Moderate flow rate can promote fish movement, while appropriate physical activity enhances blood circulation and accelerates metabolism, and this is beneficial for the overall health of fish. Suitable flow rates encourage aerobic swimming, which helps regulate blood cholesterol and triglyceride levels, as well as reduce oxidative stress levels [8]. As the flow rate increases, the locomotion of fish gradually transitions from aerobic exercise to anaerobic exercise. During this phase, the liver breaks down glycogen to produce lactic acid, resulting in a significant increase in lactic acid concentration in the blood [9]. At the same time, the concentration of lactic acid in muscles rises sharply, leading to the production of lactate ions and H+, which accelerates blood acidosis. This acidosis disrupts the internal balance of the organism, causing fish to experience fatigue more rapidly and consequently diminishing their physical performance and metabolic rate [10]. If fish are subjected to prolonged periods of rapid anaerobic swimming in high-flow-rate environments, their nutritional energy reserves will be significantly depleted. In our previous studies, we have found that fish’s weight decreases quickly (about 40% drop) after a 21-day period of starvation due to their fast swimming ability (non-stop swimming) [2]. Our research has also revealed a decrease in crude lipid and crude protein of approximately 40% and 10%, respectively, after six days of starvation [11]. These studies all indicated that this fish would quickly deplete their nutrient reserves in short-term energy-consuming activities. Therefore, the flow rate is likely to have a significant impact on the nutritional metabolism of silver pomfret.

To further verify this viewpoint, we set three (low, medium and high) flow rate groups for 8 weeks of silver pomfret culturing. In a previous study [12], we found that the growth performance is better in the medium group than the other two groups. And we sampled the muscle, liver and gut tissues at three groups in different stages at three flow-rate groups and sequenced the transcriptome of these tissues, then analyzed the expression and function of nutritional metabolism-related genes. In our previous studies, gut microbiota played an important role in nutritional metabolism in silver pomfret [13,14], so we sequenced 16S rRNA amplicon of gut microbiota in different stages at three flow-rate groups. We compared the gut microbiota composition in these groups and analyzed the function of these different microbiotas. The result could help understand the effect of different flow rates on silver pomfret nutritional metabolism, which provides the reference of flowing-water culture and recirculating aquaculture mode and promote the industrialization development of silver pomfret.

## 2. Materials and Methods

### 2.1. Fish Rearing and Sampling

All animal experiments were conducted in accordance with the recommendations of the National Institutes of Health Guide for the Care and Use of Laboratory Animals. The experimental protocols were approved by the Animal Care and Use Committee of Ningbo University.

Healthy juvenile silver pomfret (3 months old; initial body weight 2.05 ± 0.43 g) were reared in a recirculating aquaculture system (Qingdao Hishing Smart Equipment Co., Ltd., Qingdao, China) supplied with sand-filtered seawater. During the experiment, the water temperature was maintained at 24.0 ± 0.2 °C, pH at 8.11 ± 0.10, dissolved oxygen at 6.11 ± 0.71 mg/L, and salinity at 21.0 ± 0.5‰. Fish were fed a commercial diet (Sai Fengnian Floating Feed 2#; Ningbo Tianbang Feed Technology Co., Ltd., Ningbo, China) three times daily at a total ration of 3–5% of body weight. Before the formal experiment, all fish were acclimated for 1 week under a jet flow rate of 100 L/h, during which no mortality was observed and normal swimming and feeding behavior were maintained.

After acclimation, fish were randomly assigned to three jet flow-rate treatments: 400 L/h (low-flow group, L), 600 L/h (medium-flow group, M), and 800 L/h (high-flow group, H). Each treatment included four replicate tanks, with 500 fish stocked per tank. The system permitted precise regulation of the jet flow rate. In our previous study, using the same experimental system and flow-rate design, we first conducted a 1-week short-term trial to evaluate hydrodynamic characteristics together with fish behavioral and culture performance responses, including swimming behavior, feeding rate, the accumulation of residual feed and feces in the tanks, specific growth rate (SGR), feed conversion ratio (FCR), and survival rate (SGR: 3.47% in L group, 3.57% in M group, 2.65% in H group; FCR: 1.13 in L group, 1.32 in M group, 0.96 in H group; survival rate: 83.8% in L group, 87.4% in M group, 67.13% in H group) and then conducted an 8-week long-term culture trial under the same three flow-rate conditions to further analyze growth performance and nutritional metabolism [12]. In that study, growth-related traits, including body weight, total length, body length, fork length, body height, and condition factor, were measured at D0, D1 (first stage), and D2 (second stage). Fish reared at 0.4 and 0.6 m^3^/h generally showed better growth trends than those reared at 0.8 m^3^/h, although no significant differences were detected among the three groups in body weight, body length, fork length, or body height. The condition factor further indicated that fish in the two lower-flow groups exhibited a relatively plumper body condition than those in the high-flow group. Among the tested conditions, the 0.6 m^3^/h treatment showed the most favorable overall culture performance and was identified as the most suitable flow condition for juvenile silver pomfret during the rapid-growth stage, based on short-term performance indices (SGR, FCR, and survival rate) together with feeding-related performance, residual feed and fecal discharge, growth tendency, and nutritional metabolism [12]. Therefore, the present study was conducted under these previously characterized flow-rate conditions used as the experimental background for subsequent transcriptomic and gut microbiota analyses [12].

### 2.2. RNA-Sequence

#### 2.2.1. RNA Quantification and Qualification

Total RNA was extracted from liver, kidney, and spleen tissues collected at 96 h using Total RNA extraction Kit (Solarbio, Cat: R1200, Beijing Solarbio Science & Technology Co., Ltd., Beijing, China). RNA quality was preliminarily evaluated by 1% agarose gel electrophoresis and further assessed using a NanoPhotometer^®^ spectrophotometer (IMPLEN, Westlake Village, CA, USA). RNA concentration was measured using the Qubit^®^ RNA Assay Kit in conjunction with the Qubit^®^ 2.0 Fluorometer (Life Technologies, Carlsbad, CA, USA). RNA integrity was further evaluated using the RNA Nano 6000 Assay Kit on the Agilent 2100 Bioanalyzer system (Agilent Technologies, Santa Clara, CA, USA).

#### 2.2.2. Library Preparation for Transcriptome Sequencing

Sequencing libraries were constructed using the NEBNext^®^ Ultra™ RNA Library Prep Kit for Illumina^®^ (NEB, Ipswich, MA, USA), following the manufacturer’s instructions. Index codes were incorporated to distinguish individual samples. Briefly, mRNA was first enriched from total RNA using poly-T oligo-attached magnetic beads and then fragmented under elevated temperature in NEBNext First-Strand Synthesis Reaction Buffer (5×) containing divalent cations. First-strand cDNA was synthesized using random hexamer primers and M-MuLV Reverse Transcriptase (RNase H−), followed by second-strand cDNA synthesis using DNA Polymerase I and RNase H. The resulting double-stranded cDNA fragments were end-repaired to generate blunt ends, and adenylation of the 3′ ends was subsequently performed to facilitate adaptor ligation. NEBNext adaptors with hairpin loop structures were then ligated to the cDNA fragments.

To select fragments of approximately 250–300 bp, the ligation products were purified using the AMPure XP system (Beckman Coulter, Beverly, MA, USA). Thereafter, 3 μL of USER Enzyme (NEB, USA) was added to the size-selected adaptor-ligated cDNA, followed by incubation at 37 °C for 15 min and then at 95 °C for 5 min. PCR amplification was subsequently performed using Phusion High-Fidelity DNA polymerase, universal PCR primers, and Index (X) Primer. The PCR products were purified again using the AMPure XP system, and library quality was assessed on the Agilent 2100 Bioanalyzer system. Clustering of the index-coded libraries was performed on the cBot Cluster Generation System using the TruSeq PE Cluster Kit v3-cBot-HS (Illumina, San Diego, CA, USA), according to the manufacturer’s protocol. Following cluster generation, the libraries were sequenced on the Illumina HiSeq platform to generate paired-end reads.

#### 2.2.3. Data Analysis

##### Quality Control

Raw sequencing data in FASTQ format were initially processed using in-house Perl scripts. Clean reads were obtained by removing reads containing adaptor sequences, poly-N stretches, and low-quality reads. At the same time, quality metrics including Q20, Q30, and GC content were calculated for the clean data.

##### Transcriptome Assembly and Gene Functional Annotation

De novo transcriptome assembly was performed using Trinity to generate unigenes. Functional annotation of the assembled unigenes was conducted by BLASTx searches against the NCBI non-redundant protein database (NR) with an E-value threshold of 1 × 10^−5^. In addition, BLASTx searches were performed against the Swiss-Prot database and the Clusters of Orthologous Groups (COG) database using the same E-value cutoff. Based on the NR annotations, Gene Ontology (GO) terms were assigned using the Blast2GO program. GO functional classification was then performed using the GOseq R package based on the Wallenius non-central hypergeometric distribution. KEGG pathway enrichment analysis of the annotated carp orthologs was conducted using KOBAS software (KOBAS 3.0 version).

##### Differential Expression Analysis

Gene expression levels in each sample were estimated using RSEM. Differential expression analysis between two groups was carried out using the DESeq R package (version 1.18.0). Genes with an absolute log2 fold change greater than 1 and a *p* value less than 0.05 were considered differentially expressed.

##### Functional Analysis of DEGs

To categorize gene functions into the domains of cellular component, molecular function, and biological process, Gene Ontology (GO) analysis was performed. GO annotations for differentially expressed genes (DEGs) were obtained using Blast2GO (version 3.0). Pathway mapping of the identified unigenes was performed using the Kyoto Encyclopedia of Genes and Genomes (KEGG) database. KEGG enrichment analysis was subsequently conducted to identify the potential biological functions and pathways associated with the DEGs. Heatmaps were generated using TBtools (2.420 version). [15].

##### Analysis of Differential Gene Protein Interaction Network

In order to investigate the co-expressed gene network, we used all DEGs for weighted correlation network analysis by STRING database and Cytoscape software (3.10.3 version). This analysis identified some different modules in the network. Subsequently, select the color blocks with the highest correlation among all groups of the three organizations for further analysis, and perform KEGG enrichment analysis on the DEGs in each module to determine the functional significance of genes within each module.

### 2.3. 16S rRNA Amplicon Sequencing

#### 2.3.1. DNA Extraction Library Preparation and Sequencing

Total genomic DNA was extracted using the OMEGA Bacterial DNA Kit according to the manufacturer’s instructions. DNA concentration and purity were measured using a NanoDrop 1000 spectrophotometer (Thermo Fisher Scientific, Wilmington, DE, USA). Based on the quantified DNA concentration, samples were diluted to 1 ng/μL with sterile water.

The target region of the 16S rRNA gene was amplified using barcoded universal primers 515F (5′-GTGCCAGCMGCCGCGG-3′) and 806R (5′-GGACTACHVGGGTWTCTAAT-3′). PCR amplification was performed in a reaction mixture containing 15 μL of Phusion^®^ High-Fidelity PCR Master Mix (New England Biolabs, Ipswich, MA, USA), 0.2 μL each of forward and reverse primers, and 10 ng of template DNA. The PCR conditions were as follows: initial denaturation at 98 °C for 1 min; 30 cycles of denaturation at 98 °C for 10 s, annealing at 50 °C for 30 s, and extension at 72 °C for 30 s; followed by a final extension at 72 °C for 5 min. The quality and concentration of PCR products were assessed using the Agilent 5400 Fragment Analyzer system.

Sequencing libraries were constructed using the TruSeq^®^ DNA PCR-Free Sample Preparation Kit (Illumina, San Diego, CA, USA), with index codes added to each sample. Library quality was evaluated using the Qubit^®^ 2.0 Fluorometer (Thermo Fisher Scientific, USA) and the Agilent 2100 Bioanalyzer system. Qualified libraries were subsequently sequenced on an Illumina platform to generate paired-end reads.

#### 2.3.2. Functional Prediction with FAPROTAX

To infer potential ecological functions of the intestinal prokaryotic communities, functional annotation was performed using FAPROTAX (Functional Annotation of Prokaryotic Taxa) based on the taxonomic profiles derived from 16S rRNA gene sequencing data. Briefly, the ASV/OTU abundance table with taxonomic assignments was mapped to the FAPROTAX database to obtain predicted functional groups associated with carbon, nitrogen, sulfur cycling and other ecologically relevant metabolic processes [16]. The relative abundances of predicted functions were calculated for each sample and compared among treatments using one-way analysis of variance (ANOVA) followed by Tukey’s HSD post hoc test. Significance was set at *p* < 0.05.

#### 2.3.3. Data Analysis

Equimolar amounts of amplicons from each sample were pooled and sequenced in a single run on an Illumina platform. Raw 16S rRNA gene sequencing data were processed using QIIME2 (version 2026.4) [17]. Briefly, primer sequences were trimmed using the Cutadapt plugin (version 5.2) [18]. High-quality representative sequences were denoised and amplicon sequence variant (ASV) tables were generated using DADA2 (version 1.34.0) [19]. Taxonomic assignment of representative sequences was performed against the Silva 16S rRNA database (version 138.2). Non-bacterial ASVs, as well as sequences assigned to chloroplasts and archaea, were removed from the dataset. Rare ASVs with fewer than 20 reads were also excluded. To account for unequal sequencing depth across samples, all samples were rarefied to the same sequencing depth prior to downstream analyses.

#### 2.3.4. Statistical Analysis

Unless otherwise specified, all statistical analyses were conducted in R version 4.2.2 using the vegan package [20]. Tukey’s honestly significant difference (HSD) test was used to assess significant differences among groups, with a significance threshold of 0.05 [21]. Permutational multivariate analysis of variance (PERMANOVA) was further performed to evaluate the contribution of different conditions to microbiota variation [22]. Microbial beta diversity was assessed based on Bray–Curtis distance matrices and visualized by constrained principal coordinates analysis (CPCoA). Shared and unique ASVs between flatulence-affected and healthy fish were identified using the VennDiagram package. Differentially abundant ASVs between flatulence-affected and healthy fish were identified using the edgeR package. ASVs with an FDR-adjusted *p* value < 0.05 and log2(fold change) > 1 or <−1 were considered significantly enriched or depleted, respectively. SourceTracker was used to infer the origins of gut microbial communities [23]. Community similarity was evaluated using Jaccard distance and the cohesion algorithm [24]. Microbial co-occurrence network analysis and correlation analysis were performed using ggClusterNet [25]. In addition, PICRUSt2 was used to predict the functional potential of the metagenome based on 16S rRNA gene data [26]. Differences in PICRUSt2-predicted functions between flatulence-affected and healthy fish were assessed using the Wilcoxon test. Metabolic pathways were visualized using iPath (Interactive Pathways Explorer) (https://pathways.embl.de/) [27].

## 3. Results

### 3.1. Transcriptome Analysis of Silver Pomfret at Varying Flow Rates

#### 3.1.1. Transcriptome Assembly, Annotation and Quality Assessment

In 54 libraries, a total of 456.84 Mb of Clean Data was obtained, with Clean Data of over 6.5 Mb for all samples and a Q30 base percentage of over 95.32% (Table S1). A total of 44,502 unigenes were detected, and the length distribution showed that most of the unigenes fell in the range of 200–500 bp, followed by those in the range of 500–1000 bp and 1–1.5 kbp (Table 1, Figure S1A). A total of 92,186 transcripts were detected, and the length distribution was similar to that of the unigenes (Table 1, Figure S1B). The unigenes and transcripts were relatively evenly distributed among the various groups (Figure S1C,D). The unigenes were annotated by aligning them with public protein databases such as NR-NCBI, Swiss-Prot, KEGG, COG, and GO (Table 2), which were annotated by NR-NCBI (50.93%), followed by COG (44.60%), Swiss-Prot (39.80%) and KEGG (37.53%).

The correlation R2 of gene expression was less than 1, ranging from 0.003 to 0.993, which indicated a high similarity in gene expression among samples within the same group, while significant differences exist between groups (Figure S2A). Principal Component Analysis (PCA) successfully distinguished different tissues of silver pomfret under varying flow rates, with the first two principal components accounting for 33.82% and 24.47% of the variance between samples, respectively (Figure S2B). Furthermore, Venn diagram analysis demonstrated that the number of shared genes is highest in muscle tissue and lowest in the intestine (Figure S2C–E).

#### 3.1.2. DEG Analysis

A total of 6477 DEGs were obtained, including 3568 upregulated genes and 2909 downregulated genes (Figure S3A). In liver tissue (Figure S3B), there are 34 differentially expressed genes between the D1_6L and D1_4L groups, including 12 upregulated and 22 downregulated genes; there are 46 differentially expressed genes between D1_8L and D1_4L groups, including 26 upregulated and 20 downregulated genes; there are 47 differentially expressed genes between D1_8L and D1_6L groups, including 31 upregulated and 16 downregulated genes; there are 68 differentially expressed genes between D2_6L and D2_4L groups, including 29 upregulated and 39 downregulated genes; there are 21 differentially expressed genes between D2_8L and D2_4L groups, including 6 upregulated and 15 downregulated genes; there are 30 differentially expressed genes between D2_8L and D2_6L groups, including 12 upregulated and 18 downregulated genes. In intestinal tissue (Figure S3C), there were 206 differentially expressed genes between the D1_6G and D1_4G groups, including 93 upregulated and 113 downregulated genes; there are 72 differentially expressed genes between D1_8G and D1_4G groups, including 49 upregulated and 23 downregulated genes; there are 320 differentially expressed genes between the D1_8G and D1_6G groups, including 151 upregulated and 169 downregulated genes; there are 45 differentially expressed genes between D2_6G and D2_4G groups, including 28 upregulated and 17 downregulated genes; there are 582 differentially expressed genes in the D2_8G vs. D2_24G group, including 412 upregulated and 170 downregulated genes; there are 488 differentially expressed genes between D2_8G and D2_6G groups, including 262 upregulated and 226 downregulated genes. In muscle tissue (Figure S3D), there are 378 differentially expressed genes between D1_6M and D1_4M groups, including 287 upregulated and 91 downregulated genes; there are 1513 differentially expressed genes between D1_8M and D1_4M groups, including 696 upregulated and 817 downregulated genes; there are 972 differentially expressed genes between D1_8M and D1_6M groups, including 529 upregulated and 443 downregulated genes; there are 84 differentially expressed genes between D2_6M and D2_4M groups, including 42 upregulated and 42 downregulated genes; there are 338 differentially expressed genes between D2_8M and D2_4M groups, including 194 upregulated and 144 downregulated genes; there are 1233 differentially expressed genes between D2_8M and D2_6M groups, including 709 upregulated and 524 downregulated genes.

#### 3.1.3. GO and KEGG Analysis of DEG

**Figure 1:**
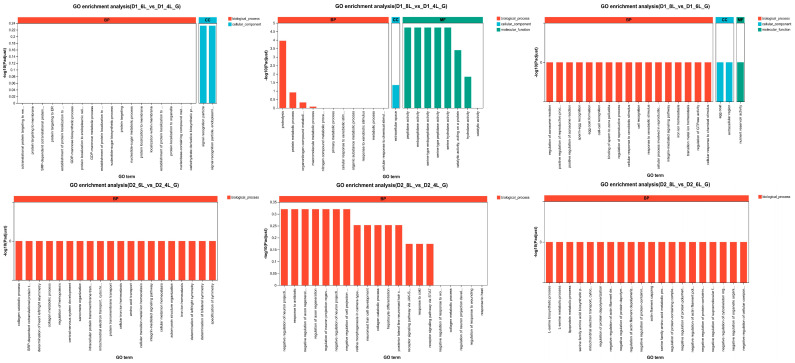
High enriched GO terms in liver tissues of silver pomfret at varying flow rates. D1_4/6/8L: liver for 400/600/800 L/h group at 4 weeks (first stage); D1_4/6/8G: gut for 400/600/800 L/h group at 4 weeks (first stage); D1_4/6/8M: muscle for 400/600/800 L/h group at 4 weeks (first stage); D2_4/6/8 L: liver for 400/600/800 L/h group at 8 weeks (second stage); D2_4/6/8G: gut for 400/600/800 L/h group at 8 weeks (second stage); D2_4/6/8M: muscle for 400/600/800 L/h group at 8 weeks (second stage). Complete GO description is supplied in Supplemental Materials File S1.

**Figure 2:**
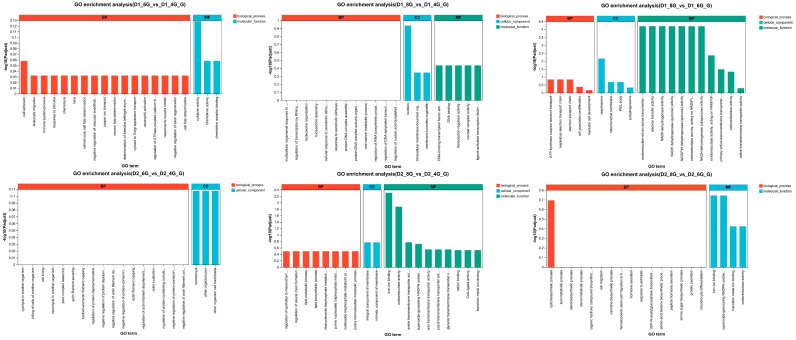
High enriched GO terms in gut tissues of silver pomfret at varying flow rates. D1_4/6/8L: liver for 400/600/800 L/h group at 4 weeks (first stage); D1_4/6/8G: gut for 400/600/800 L/h group at 4 weeks (first stage); D1_4/6/8M: muscle for 400/600/800 L/h group at 4 weeks (first stage); D2_4/6/8 L: liver for 400/600/800 L/h group at 8 weeks (second stage); D2_4/6/8G: gut for 400/600/800 L/h group at 8 weeks (second stage); D2_4/6/8M: muscle for 400/600/800 L/h group at 8 weeks (second stage). Complete GO discription is supplied in Supplemental Material File S1.

**Figure 3:**
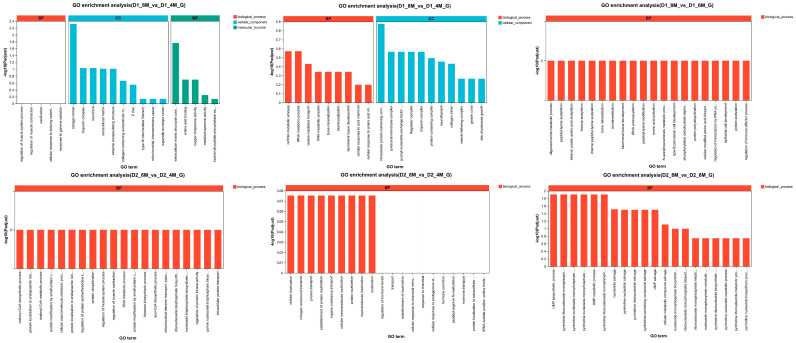
High enriched GO terms in muscle tissues of silver pomfret at varying flow rates. D1_4/6/8L: liver for 400/600/800 L/h group at 4 weeks (first stage); D1_4/6/8G: gut for 400/600/800 L/h group at 4 weeks (first stage); D1_4/6/8M: muscle for 400/600/800 L/h group at 4 weeks (first stage); D2_4/6/8 L: liver for 400/600/800 L/h group at 8 weeks (second stage); D2_4/6/8G: gut for 400/600/800 L/h group at 8 weeks (second stage); D2_4/6/8M: muscle for 400/600/800 L/h group at 8 weeks (second stage). Complete GO discription is supplied in Supplemental Material File S1.

The differentially expressed genes (DEGs) identified in the study were found to be enriched in the three primary categories of Gene Ontology [28], namely biological process, cellular component, and molecular function (Table S2). Specifically, in the first stage (D1), in the comparison of D1_6L vs. D1_4L, D1_8L vs. D1_4L and D1_8L vs. D1_6L (Figure 1), significant enrichment of DEGs was observed in GO terms associated with signal recognition particle, peptidase activity, regulation of acrosome reaction; in the comparison of D1_6G vs. D1_4G, D1_8G vs. D1_4G, D1_8G vs. D1_6G (Figure 2), the DEGs were significantly enriched in GO terms related to cytokine activity, nucleus, oxidoreduction-driven active transmembrane transporter activity; in the comparison of D1_6M vs. D1_4M, D1_8M vs. D1_4M, D1_8M vs. D1_6M (Figure 3), the DEGs were significantly enriched in GO terms related to collagen trimer, intracellular protein-containing complex, oligosaccharide metabolic process. In second stage (D2), in the comparison of D2_6L vs. D2_4L, D2_8L vs. D2_4L and D2_8L vs. D2_6L (Figure 1), significant enrichment of DEGs was observed in GO terms associated with collagen catabolic process, negative regulation of neuron projection regeneration, and L-serine biosynthetic process; in the comparison of D2_6G vs. D2_4G, D2_8G vs. D2_4G, D2_8G vs. D2_6G (Figure 2), the DEGs were significantly enriched in GO terms related to nematocyst, iron ion binding; in the comparison of D2_6M vs. D2_4M, D2_8M vs. D2_4M, D2_8M vs. D2_6M (Figure 3), the DEGs were significantly enriched in GO terms related to malonyl-CoA biosynthetic process, cellular localization, UMP biosynthetic process.

**Figure 4:**
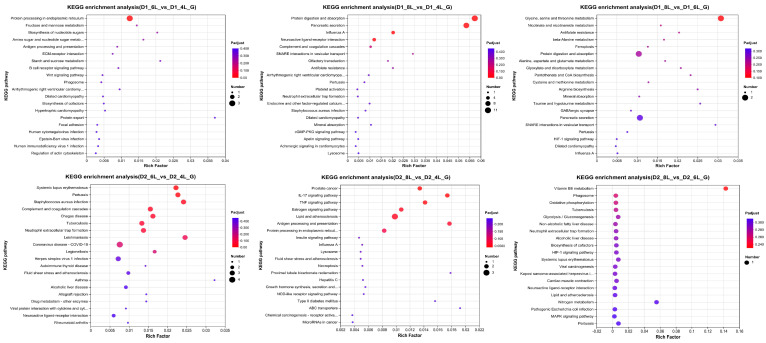
High enriched KEGG pathways in liver tissues of silver pomfret at varying flow rates. D1_4/6/8L: liver for 400/600/800 L/h group at 4 weeks (first stage); D1_4/6/8G: gut for 400/600/800 L/h group at 4 weeks (first stage); D1_4/6/8M: muscle for 400/600/800 L/h group at 4 weeks (first stage); D2_4/6/8 L: liver for 400/600/800 L/h group at 8 weeks (second stage); D2_4/6/8G: gut for 400/600/800 L/h group at 8 weeks (second stage); D2_4/6/8M: muscle for 400/600/800 L/h group at 8 weeks (second stage).

**Figure 5:**
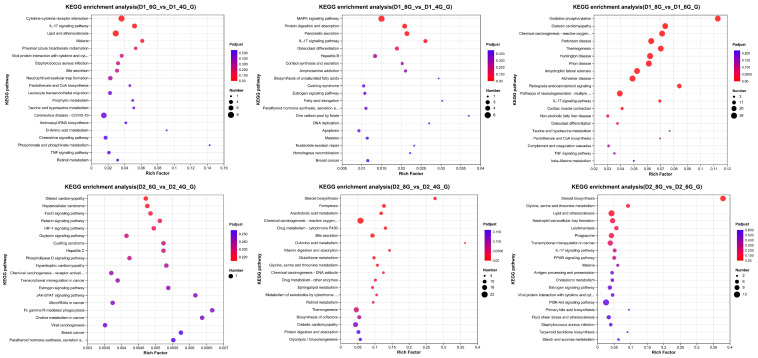
High enriched KEGG pathways in gut tissues of silver pomfret at varying flow rates. D1_4/6/8L: liver for 400/600/800 L/h group at 4 weeks (first stage); D1_4/6/8G: gut for 400/600/800 L/h group at 4 weeks (first stage); D1_4/6/8M: muscle for 400/600/800 L/h group at 4 weeks (first stage); D2_4/6/8 L: liver for 400/600/800 L/h group at 8 weeks (second stage); D2_4/6/8G: gut for 400/600/800 L/h group at 8 weeks (second stage); D2_4/6/8M: muscle for 400/600/800 L/h group at 8 weeks (second stage).

**Figure 6:**
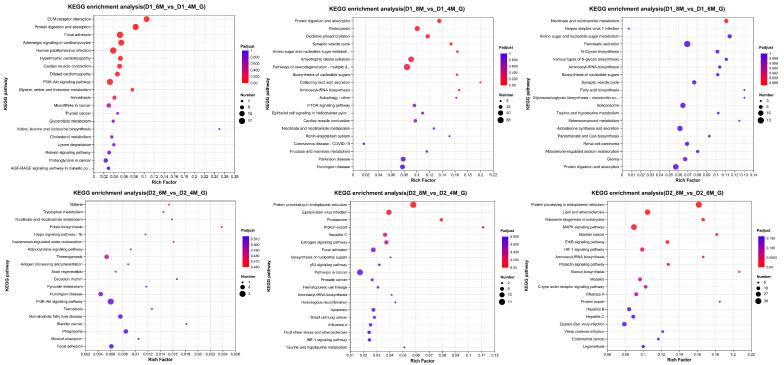
High enriched KEGG pathways in muscle tissues of silver pomfret at varying flow rates. D1_4/6/8L: liver for 400/600/800 L/h group at 4 weeks (first stage); D1_4/6/8G: gut for 400/600/800 L/h group at 4 weeks (first stage); D1_4/6/8M: muscle for 400/600/800 L/h group at 4 weeks (first stage); D2_4/6/8 L: liver for 400/600/800 L/h group at 8 weeks (second stage); D2_4/6/8G: gut for 400/600/800 L/h group at 8 weeks (second stage); D2_4/6/8M: muscle for 400/600/800 L/h group at 8 weeks (second stage).

Regarding KEGG enrichment, the top five pathways in the three examined tissues are presented in Table S3. Specifically, in first stage (D1), in the comparison of D1_6L vs. D1_4L, D1_8L vs. D1_4L and D1_8L vs. D1_6L (Figure 4), significant enrichment of DEGs was observed in KEGG pathways associated with protein processing in endoplasmic reticulum, protein digestion and absorption, glycine, serine and threonine metabolism; in the comparison of D1_6G vs. D1_4G, D1_8G vs. D1_4G, D1_8G vs. D1_6G (Figure 5), the DEGs were significantly enriched in KEGG pathways related to cytokine–cytokine receptor interaction, MAPK signaling pathway, oxidative phosphorylation; in the comparison of D1_6M vs. D1_4M, D1_8M vs. D1_4M, D1_8M vs. D1_6M (Figure 6), the DEGs were significantly enriched in KEGG pathways related to ECM–receptor interaction, protein digestion and absorption, nicotinate and nicotinamide metabolism. In the second stage (D2), in the comparison of D2_6L vs. D2_4L, D2_8L vs. D2_4L and D2_8L vs. D2_6L (Figure 4), significant enrichment of DEGs was observed in KEGG pathways associated with systemic lupus erythematosus, prostate cancer, and vitamin B6 metabolism; in the comparison of D2_6G vs. D2_4G, D2_8G vs. D2_4G, D2_8G vs. D2_6G (Figure 5), the DEGs were significantly enriched in KEGG pathways related to dilated cardiomyopathy, steroid biosynthesis, steroid biosynthesis; in the comparison of D2_6M vs. D2_4M, D2_8M vs. D2_4M, D2_8M vs. D2_6M (Figure 6), the DEGs were significantly enriched in KEGG pathways related to malaria and protein processing in endoplasmic reticulum.

#### 3.1.4. Analysis of Nutritional Metabolism Related DEG

**Figure 7:**
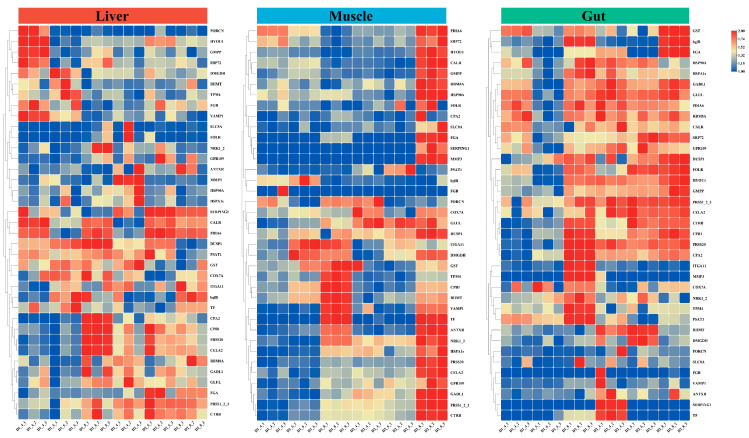
Heatmap expression level analysis of DEG related to nutritional material synthesis, metabolism, and oxidation–reduction reactions in liver, gut and muscle tissues of silver pomfret at varying flow rates. D1_4/6/8L: liver for 400/600/800 L/h group at 4 weeks (first stage); D1_4/6/8G: gut for 400/600/800 L/h group at 4 weeks (first stage); D1_4/6/8M: muscle for 400/600/800 L/h group at 4 weeks (first stage); D2_4/6/8 L: liver for 400/600/800 L/h group at 8 weeks (second stage); D2_4/6/8G: gut for 400/600/800 L/h group at 8 weeks (second stage); D2_4/6/8M: muscle for 400/600/800 L/h group at 8 weeks (second stage).

We selected the DEGs from high enriched KEGG pathway related to nutritional material synthesis, metabolism, and oxidation–reduction reactions and analyzed the expression level of them through heatmap visualization (Figure 7). The results indicated that genes associated with material synthesis, such as HYOU1, PDIA6, ITGA11, MMP3, and COX7A, exhibited higher expression levels in muscle and gut tissues within the D2_8 group. In contrast, these genes showed significantly elevated expression in liver tissue during the D1_4 group. Genes involved in metabolism, including CPA2, CPB1, SERPING1, GMPP, and GPR109, demonstrated peak expression levels across all three tissues in both the D1_8 and D2_8 groups. This pattern aligns with the expression trends observed for genes related to material synthesis. Furthermore, genes pertinent to oxidation–reduction reactions such as GLUL, DUSP1, GST, HSP90A, and HSPA1s displayed heightened expression levels in liver and gut tissues during the D1_6/8 and D2_6/8 groups.

#### 3.1.5. WGCNA Analysis

**Figure 8:**
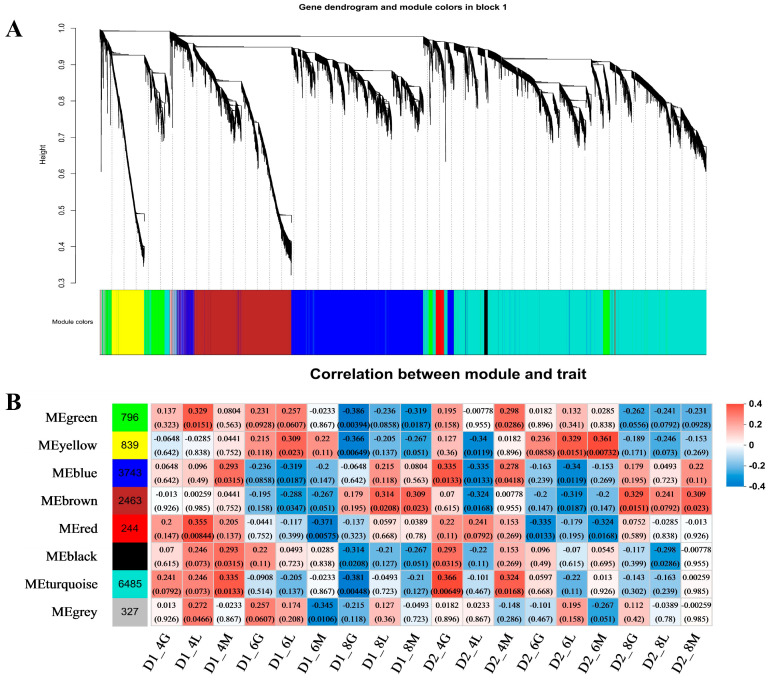
Hierarchical clustering construction of gene modules and correlation heatmaps between samples and modules. (**A**) Gene dendrogram and module colors in block 1; (**B**) Correlation between module and trait.

**Figure 9:**
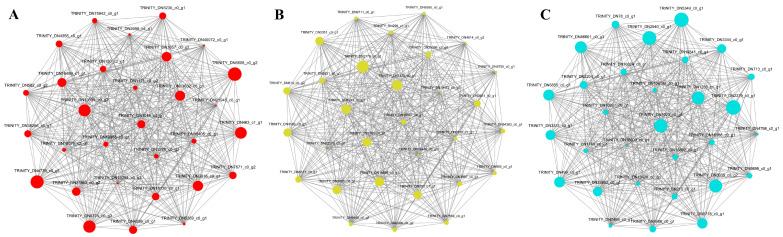
Network diagram of the highest correlation color blocks in liver (**A**), muscle (**B**), and gut (**C**) tissues.

To investigate the co-expression gene network of silver pomfret under different flow rates, we used 6477 DEGs for WGCNA analysis. This analysis identified eight different modules in the network (Figure 8). We then selected the three most highly correlated clusters from all tissues for further analysis (Figure 9) and performed KEGG enrichment analysis on the DEGs in each module to determine the functional significance of genes within each module. The liver tissue selected the red module: the co-expressed genes in this module were significantly enriched for insulin secretion, lipolysis in adipocytes, and stomach acid secretion. The muscle tissue selected the yellow module: the co-expressed genes in this module were significantly enriched for saliva secretion, regulation of actin cytoskeleton, and glycine, serine and threonine metabolism. The intestinal tissue selected the turquoise module: lysine degradation, HIF-1 signaling pathway, and MAPK signaling pathway.

### 3.2. The Effect of Different Flow Rates on the Gut Microbiota of Silver Pomfret

#### 3.2.1. The α and β Diversity 16S rRNA Sequencing Results

**Figure 10:**
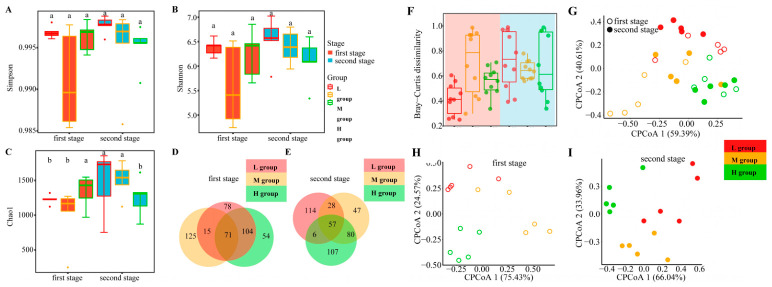
The differences in alpha and β diversity at different flow rates during different stages. (**A**): Differences in Simpson index at different flow rates in different stages. (**B**): Differences in Shannon index at different flow rates in different stages. (**C**): Differences in Chao1 index at different flow rates in different stages. (**D**,**E**): The number of differential ASVs at different flow rates in different stages. (**F**): The difference in BC distance at different flow rates in different stages; (**G**–**I**): CPCoA at different flow rates at different stages (first stage: 4 weeks; second stage: 8 weeks).

A total of 1,073,799 high-quality and classifiable reads were obtained in this study, ranging from 26,510 to 49,070 reads per sample. These reads corresponded to 2404 amplicon sequence variants (ASVs) across all samples. Then, we compared the alpha diversity of each group. There is no significant difference between different flow velocities in the first stage on the Shannon index and Simpson index (Figure 10A,B, Table S4). The Chao1 index with a flow rate of 8 units in the first stage is higher than the other two low-flow-rate groups (Figure 10C, Table S4). In the second stage, as the flow rate increases, the Shannon index, Simpson index, and Chao1 index continuously decrease; in the first stage, there were 78 unique ASVs at four flow rates, 125 unique ASVs in M group, 54 unique ASVs at eight flow rates, and 71 shared ASVs at different flow rates. In the second stage, there were 114 unique ASVs in L group, 47 unique ASVs at six flow rates, 107 unique ASVs at H group, and 57 shared ASVs at different flow rates. Overall, the flow rate in the second stage of treatment has a significant impact on the alpha diversity of microbial communities (Figure 10D,E, Table S4).

Based on the analysis of species similarity using Bray–Curtis distance, we found that in the first stage of treatment, the species with M group had the lowest similarity and the largest difference. However, in the second stage of treatment, the species with M group had the highest similarity and the smallest difference (Figure 10F). Through the analysis based on BC distance CPCoA, it can be found that there are significant differences in gut microbiota at the same flow rate under different stages (Figure 10G–I). Similarly, there are significant differences in the gut microbiota at different flow rates under the same stages (Figure 10G–I).

#### 3.2.2. Bacterial Composition and Difference in Silver Pomfret at Different Flow Rates

**Figure 11:**
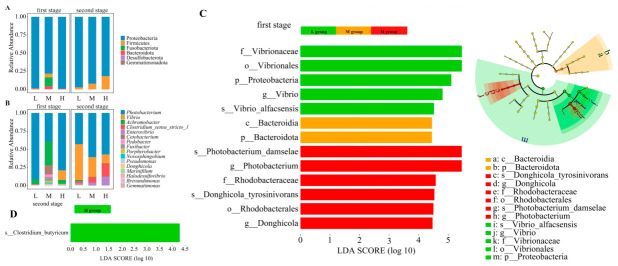
Bacterial composition and difference at different flow rates in different stages. (**A**): Bacterial composition at phylum levels. (**B**): Bacterial composition at genus level. (**C**,**D**): LEfSe analysis of bacterial at different flow rates in first and second stages (first stage: 4 weeks; second stage: 8 weeks).

In terms of phylum level, the most dominant phylum in all samples was *Proteobacteria*, accounting for 78.4% to 99.2% of the total microbial population. At the first stage, the relative abundances of Firmicutes and Fusobacteriota were significantly higher in the M group than in the other two groups. In addition, the relative abundance of Firmicutes increased with increasing flow rate, while the relative abundance of Bacteroidota was highest in the H group (Figure 11A). At the genus level, differences in microbial composition were analyzed (Figure 11B). *Photobacteria* (69.9%) and *Achromobacter* (15.1%) are dominant in the first stage. Among them, *Photobacteria* has an average abundance of 91.4% in L group, 39.2% in M group, and 79.2% in H group. The average abundance of *Achromobacter* in L group accounts for 6.8%, the average abundance in M group accounts for 32.8%, and the average abundance in H group accounts for 5.5%. The advantages in the second stage processing are *Photobacteria* (53.8%) and *Vibrio* (29.3%). Among them, *Photobacteria* has an average abundance of 43.1% in L group, 60.9% in M group, and 57.3% in H group. The average abundance of *Achromobacter* in L group accounts for 49.1%, the average abundance in M group accounts for 27.0%, and the average abundance in H group accounts for 11.7%. In the second stage, we found that the average abundance of Clostridium-sensu-stricto_1 and Enterovibrio increased with the increase in flow rate. In order to identify the significant differences in gut microbiota under different flow rates, we conducted LEfSe analysis and constructed a taxonomic evolutionary tree based on it. At the first stage (Figure 11C), the abundance of *Photobacterium damselae* significantly increased in L group compared to other groups. In the M group, the abundance of Bacteroids in the Bacteroidota phylum significantly increased. The abundance of *Vibrio alfacsensis* in the Vibrio genus is significant more in H group than other groups. At the second stage (Figure 11D), only significant differences were found in the abundance of clostridium–butyricum at H group among the three groups.

#### 3.2.3. Stability and Community Assembly Analysis at Different Flow Rates

Based on Spearman correlation symbiotic network analysis for all groups (Figure S4), in the first stage, the number of network nodes and edges continued to increase with the increase in flow rate, and in the second stage, the number of edges also continued to increase with the increase in flow rate, while the number of nodes decreased. Through further analysis of the network, in the first stage, the stability of the network was highest in M group, while in the second stage, the stability increases with the increase in flow rate.

Using the Sloan neutral model to compare the neutral process of gut microbiota assembly at different flow rates during different periods, the fitting degree in the first stage decreases with increasing flow rate (Figure S5A). The fitting degree is best in L group, but the proportion of ASV that conforms to neutral processes is the lowest. The Nm (11,919) value in L group was higher than the other two groups, indicating that species diffusion at this group was significantly higher than in the other groups. The fitting degree in the second stage is relatively low, with the proportion of ASVs that conform to neutral processes being 85.8% (L group), 47.3% (M group), and 80% (H group), respectively (Figure S5B).

Through iCAMP, we found that there is a significant heterogeneous selection effect in the first stage in M group. In the second stage, the effect of drift decreases with increasing flow rate, while the effect of diffusion limitation increases. Overall, in the first stage, different groups showed significant differences in the community construction process, with L and M groups being greatly affected by the selection process, and H group being jointly affected by the random and selection processes. In the second stage, the community construction of all groups tends towards a random process, and the relative contribution of the drift process increases. With the passage of stages, the relative abundance and frequency distribution of species have also undergone significant changes, especially the proportion of high-frequency species in L and H groups has significantly decreased (Figure S5C,D).

#### 3.2.4. The Function Prediction of Microbiota at Different Flow Rates

**Figure 12:**
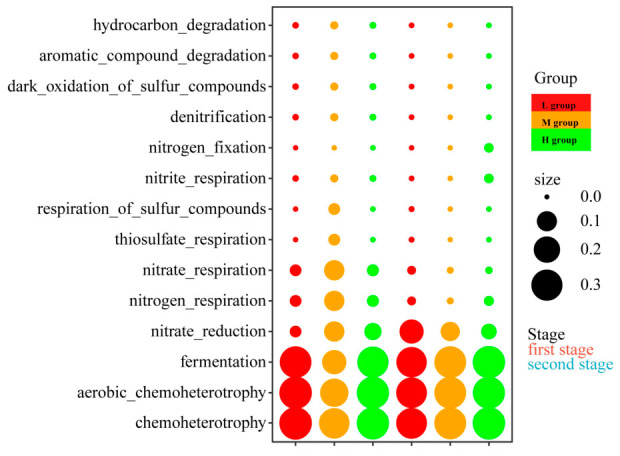
Microbial community function prediction at different flow rates (first stage: 4 weeks; second stage: 8 weeks).

FAPROTAX predicted the potential functions of gut microbiota between different groups, and fermentation and chemoheterotrophy showed relatively high relative abundance in all groups, especially in the first stage, where the abundance of these two functions was relatively high in L group. Aerobic chemoheterotrophy also showed high abundance in all groups, especially in M and H groups. The abundance of nitrate respiration, sulfide respiration, denitrification, nitrogen fixation, and other functions is relatively low in each group. In most functions, the abundance in M and H groups is relatively high, indicating that these groups may have higher functional diversity. The difference in functional abundance between different days is not significant, indicating that time has a relatively small impact on the functions of this microbiota (Figure 12).

## 4. Discussion

### 4.1. Impact of Water Flow Rate on the Growth Performance of Silver Pomfret

This study found that the growth performance of silver pomfret in the moderate-flow-rate group (600 L/h) was significantly better than that in the low- and high-flow-rate groups (400 L/h and 800 L/h respectively). This is consistent with previous research, indicating that an appropriate water flow rate can promote fish movement, enhance metabolism, and thereby improve growth efficiency [29,30]. The moderate flow rate likely provides an optimal hydrodynamic environment for silver pomfret, allowing them to remain active without excessive energy expenditure. Specifically, the moderate flow rate may stimulate aerobic swimming, which enhances blood circulation and nutrient utilization, leading to improved growth rates and feed conversion efficiency. This finding aligns with studies on other fish species [31,32], where moderate water flow has been shown to optimize energy expenditure and growth performance.

Although the high-flow-rate group exhibited increased fish movement, excessively high flow rates may induce anaerobic exercise in silver pomfret, leading to lactate accumulation, blood acidosis, and ultimately causing fatigue and reduced metabolic rates [33,34]. This aligns with our observation of weight loss in silver pomfret under high-flow-rate conditions. The transition from aerobic to anaerobic metabolism under high flow rates likely results in the depletion of energy reserves, as evidenced by the significant reduction in body weight [35,36]. These findings underscore the importance of avoiding excessively high flow rates in aquaculture to prevent metabolic stress and ensure optimal growth.

### 4.2. Impact of Water Flow Rate on the Nutritional Metabolism of Silver Pomfret

Transcriptome analysis revealed significant differences in the expression of genes related to nutritional metabolism under different flow rates. Silver pomfret in the moderate-flow-rate group showed higher expression levels of genes associated with material synthesis and metabolism, such as HYOU1, PDIA6, and ITGA11, in muscle and intestinal tissues. The upregulation of these genes may promote the synthesis of proteins and lipids, thereby enhancing growth performance. For instance, HYOU1, a hypoxia-upregulated gene, plays a critical role in protein folding and cellular stress responses [37], while PDIA6 is involved in disulfide bond formation and redox regulation, both of which are essential for maintaining cellular homeostasis under varying environmental conditions [38]. ITGA11, on the other hand, mediates cell–matrix interactions [39], potentially enhancing tissue repair and growth under optimal flow conditions.

Silver pomfret in the moderate-flow-rate group exhibited higher expression levels of genes related to redox reactions, such as GLUL, DUSP1, and GST, in liver and intestinal tissues. The upregulation of these genes may help silver pomfret better cope with oxidative stress, maintain homeostasis, and improve survival rates. GLUL (glutamine synthetase) is crucial for nitrogen metabolism and detoxification [40], while DUSP1 (dual-specificity phosphatase 1) regulates stress-responsive signaling pathways [41,42]. GST (glutathione S-transferase) plays a pivotal role in detoxifying reactive oxygen species [43], thereby protecting cells from oxidative damage. The enhanced expression of these genes under moderate flow rates suggests an adaptive mechanism to maintain cellular integrity and metabolic efficiency.

### 4.3. Impact of Water Flow Rate on the Gut Microbiota of Silver Pomfret

16S rRNA sequencing results indicated that the gut microbiota of silver pomfret in the moderate-flow-rate group exhibited the highest diversity in first culture stage (4 weeks) and stability in second stage (8 weeks). An appropriate flow rate may provide a stable environment for gut microbiota, promoting the growth of beneficial microbial communities and thereby enhancing nutrient absorption and immune capacity [44,45]. The increased diversity of gut microbiota under moderate flow rates is likely associated with improved digestive efficiency and resilience to environmental stressors. This finding is consistent with studies in other aquatic species [46], where stable microbial communities are linked to better growth and health outcomes.

FAPROTAX functional prediction showed that the gut microbiota of silver pomfret in the moderate-flow-rate group had higher abundances of functions related to fermentation and chemoheterotrophy, which may be associated with improved nutritional metabolic efficiency. Additionally, the microbial community in the moderate-flow-rate group displayed higher functional diversity, potentially further enhancing the adaptability of silver pomfret to environmental changes [47]. The enrichment of fermentation-related functions suggests enhanced breakdown of complex nutrients [48], while chemoheterotrophy indicates efficient energy extraction from organic compounds [49]. These functional adaptations likely contribute to the superior growth performance observed in the moderate-flow-rate group.

### 4.4. Practical Implications of Water Flow Rate for Silver Pomfret Aquaculture

This study demonstrates that a moderate flow rate (600 L/h) is optimal for the flow-through aquaculture of silver pomfret, significantly improving growth performance and survival rates. This finding provides important practical guidance for the large-scale farming of silver pomfret, helping to optimize aquaculture models and increase farming efficiency. By implementing moderate flow rates, aquaculturists can achieve higher yields and reduce production costs, making silver pomfret farming more economically viable.

By regulating water flow rates, the outbreak of diseases during high-temperature seasons can be effectively reduced. Silver pomfret in the moderate-flow-rate group exhibited higher expression of immune-related genes and a more stable gut microbiota, which may contribute to their stronger disease resistance [50]. The improved immune response and microbial stability under moderate flow rates likely reduce the susceptibility to pathogens, thereby minimizing the need for antibiotics and other chemical treatments. This aligns with the growing demand for sustainable and environmentally friendly aquaculture practices.

## 5. Conclusions

This study systematically analyzed the effects of different water flow rates on the growth, nutritional metabolism, and gut microbiota of silver pomfret through transcriptome and 16S rRNA amplicon sequencing. The results indicate that a moderate flow rate (600 L/h) is optimal for flow-through aquaculture of silver pomfret, significantly improving growth performance, nutritional metabolic efficiency, and disease resistance. This finding provides important theoretical and practical foundations for the large-scale farming of silver pomfret and lays a solid groundwork for its industrial development. Future research should focus on long-term effects, microbiota–host interactions, and multi-factor optimization to further advance sustainable aquaculture practices.

## Figures and Tables

**Table 1:** Assessment of transcriptome assembly.

Type	Unigene	Transcript
Total number	44,502	92,186
Total base	74,393,674	153,406,235
Largest length (bp)	28,120	28,120
Smallest length (bp)	201	201
Average length (bp)	1671.69	1664.09
N50 length (bp)	3302	2984
E90N50 length (bp)	3634	2902
Fragment mapped percent (%)	59.829	71.09
GC percent (%)	44.14	44.11
TransRate score	0.24313	0.30376
BUSCO score	C:87.8% [S:83.9%; D:3.9%]	C:95.1% [S:47.1%; D:48.0%]

**Table 2:** Summary of functional annotation of unigenes.

Databases	Unigene Number	Percent
GO	7027	15.79%
KEGG	16,702	37.53%
COG	19,848	44.60%
NR	22,664	50.93%
Swiss-Prot	17,713	39.80%
Pfam	15,894	35.72%
Total	44,502	100.00%

## Data Availability

The original contributions presented in this study are included in the article/Supplementary Material. Further inquiries can be directed to the corresponding author.

## Supplementary Materials

The following supporting information can be downloaded at: https://www.mdpi.com/article/10.3390/ani16121818/s1, Figure S1: Length distribution and unigene and transcript distribution. D1_4/6/8L: liver for 400/600/800 L/h group at 4 weeks (first stage); D1_4/6/8G: gut for 400/600/800 L/h group at 4 weeks (first stage); D1_4/6/8M: muscle for 400/600/800 L/h group at 4 weeks (first stage); D2_4/6/8 L: liver for 400/600/800 L/h group at 8 weeks (second stage); D2_4/6/8G: gut for 400/600/800 L/h group at 8 weeks (second stage); D2_4/6/8M: muscle for 400/600/800 L/h group at 8 weeks (second stage). Figure S2: Sample correlation heatmap, PCA, and inter group Venn plot between samples. D1_4/6/8L: liver for 400/600/800 L/h group at 4 weeks (first stage); D1_4/6/8G: gut for 400/600/800 L/h group at 4 weeks (first stage); D1_4/6/8M: muscle for 400/600/800 L/h group at 4 weeks (first stage); D2_4/6/8 L: liver for 400/600/800 L/h group at 8 weeks (second stage); D2_4/6/8G: gut for 400/600/800 L/h group at 8 weeks (second stage); D2_4/6/8M: muscle for 400/600/800 L/h group at 8 weeks (second stage). Figure S3: DEG analysis among different groups. D1_4/6/8L: liver for 400/600/800 L/h group at 4 weeks (first stage); D1_4/6/8G: gut for 400/600/800 L/h group at 4 weeks (first stage); D1_4/6/8M: muscle for 400/600/800 L/h group at 4 weeks (first stage); D2_4/6/8 L: liver for 400/600/800 L/h group at 8 weeks (second stage); D2_4/6/8G: gut for 400/600/800 L/h group at 8 weeks (second stage); D2_4/6/8M: muscle for 400/600/800 L/h group at 8 weeks (second stage). Figure S4: Network interaction and stability analysis at different flow rates. A/B: Network interaction; C/D/E/F: Network stability analysis. The robustness after removed random nodes. The absolute value of negative cohesion/AVD (Average Variance)/vulnerability. Figure S5: The neutral model analysis of the community assembly mechanism and quantitatively evaluates the ecological processes using iCAMP at different flow rates. C: The distribution of βNTI at different flow rates in different stages, where βNTI < −2 indicates homogeneous selection and βNTI > 2 indicates heterogeneous selection, |βNTI| < 2 indicates a neutral process. D: The proportion of community construction processes between utilization at different flow rates. Table S1: Quality analysis of transcriptome data in 54 libraries. Table S2: Top five GO terms in different comparing groups. Table S3: Top five KEGG pathways in different comparing groups. Table S4: The α diversity of silver pomfret at different flow rates. File S1: Complete GO description in Figure 1, Figure 2 and Figure 3.
