# Supplementary material for: Transcriptome and 16S rRNA Amplicon Sequencing Analysis of Nutrition Metabolism in Silver Pomfret at Varying Flow Rates
Source: Animals (Basel). 2026 Jun 12;16(12):1818. doi: 10.3390/ani16121818 (PMC13295404; doi:10.3390/ani16121818)
Supplement: Supplementary file 1 [file animals-16-01818-s001.zip › File S1.pdf]

go_id	go_type	discription
GO:0007155	BP	cell adhesion
GO:0050900	BP	leukocyte migration
GO:0002376	BP	immune system process
GO:0050896	BP	response to stimulus
GO:0006935	BP	chemotaxis
GO:0042330	BP	taxis
GO:0042671	BP	retinal cone cell fate determination
GO:1900747	BP	negative regulation of vascular endothelial growth factor signaling pathway
GO:0006825	BP	copper ion transport
GO:0048664	BP	neuron fate determination
GO:0071908	BP	determination of intestine left/right asymmetry
GO:0140820	BP	cytosol to Golgi apparatus transport
GO:0042119	BP	neutrophil activation
GO:1901894	BP	regulation of ATPase-coupled calcium transmembrane transporter activity
GO:0035994	BP	response to muscle stretch
GO:0048681	BP	negative regulation of axon regeneration
GO:0001709	BP	cell fate determination
GO:0010977	BP	negative regulation of neuron projection development
GO:0043703	BP	photoreceptor cell fate determination
GO:0070571	BP	negative regulation of neuron projection regeneration
GO:0070570	BP	regulation of neuron projection regeneration

GO:0097241	BP	hematopoietic stem cell migration to bone marrow
GO:0036230	BP	granulocyte activation
GO:1990009	BP	retinal cell apoptotic process
GO:0015783	BP	GDP-fucose transmembrane transport
GO:0015780	BP	nucleotide-sugar transmembrane transport
GO:0061298	BP	retina vasculature development in camera-type eye
GO:0034332	BP	adherens junction organization
GO:0036085	BP	GDP-fucose import into Golgi lumen
GO:0048679	BP	regulation of axon regeneration
GO:0031345	BP	negative regulation of cell projection organization
GO:0001895	BP	retina homeostasis
GO:0090480	BP	purine nucleotide-sugar transmembrane transport
GO:1902548	BP	negative regulation of cellular response to vascular endothelial growth factor stimulus
GO:0006955	BP	immune response
GO:0040011	BP	locomotion
GO:0006954	BP	inflammatory response
GO:0016477	BP	cell migration
GO:0048870	BP	cell motility
GO:0035469	BP	determination of pancreatic left/right asymmetry
GO:0035675	BP	neuromast hair cell development
GO:0035677	BP	posterior lateral line neuromast hair cell development
GO:0060876	BP	semicircular canal formation

GO:0030513	BP	positive regulation of BMP signaling pathway
GO:0031076	BP	embryonic camera-type eye development
GO:0060042	BP	retina morphogenesis in camera-type eye
GO:0097530	BP	granulocyte migration
GO:0044030	BP	regulation of DNA methylation
GO:0070365	BP	hepatocyte differentiation
GO:0030574	BP	collagen catabolic process
GO:0030593	BP	neutrophil chemotaxis
GO:0046368	BP	GDP-L-fucose metabolic process
GO:0090100	BP	positive regulation of transmembrane receptor protein serine/threonine kinase signaling pathway
GO:0071621	BP	granulocyte chemotaxis
GO:0001955	BP	blood vessel maturation
GO:0006021	BP	inositol biosynthetic process
GO:0035701	BP	hematopoietic stem cell migration
GO:0030488	BP	tRNA methylation
GO:0042350	BP	GDP-L-fucose biosynthetic process
GO:0042351	BP	'de novo' GDP-L-fucose biosynthetic process
GO:1990266	BP	neutrophil migration
GO:0002274	BP	myeloid leukocyte activation
GO:0006952	BP	defense response
GO:0032501	BP	multicellular organismal process
GO:0090136	BP	epithelial cell-cell adhesion

GO:1901019	BP	regulation of calcium ion transmembrane transporter activity
GO:0071907	BP	determination of digestive tract left/right asymmetry
GO:0097696	BP	receptor signaling pathway via STAT
GO:0007259	BP	receptor signaling pathway via JAK-STAT
GO:0019556	BP	histidine catabolic process to glutamate and formamide
GO:0019557	BP	histidine catabolic process to glutamate and formate
GO:0051924	BP	regulation of calcium ion transport
GO:0043606	BP	formamide metabolic process
GO:0006020	BP	inositol metabolic process
GO:0048264	BP	determination of ventral identity
GO:0015942	BP	formate metabolic process
GO:1903169	BP	regulation of calcium ion transmembrane transport
GO:0009605	BP	response to external stimulus
GO:0042127	BP	regulation of cell population proliferation
GO:0032412	BP	regulation of ion transmembrane transporter activity
GO:0006536	BP	glutamate metabolic process
GO:0006547	BP	histidine metabolic process
GO:0022898	BP	regulation of transmembrane transporter activity
GO:0046855	BP	inositol phosphate dephosphorylation
GO:0032409	BP	regulation of transporter activity
GO:0003140	BP	determination of left/right asymmetry in lateral mesoderm
GO:1904062	BP	regulation of cation transmembrane transport

GO:0006548	BP	histidine catabolic process
GO:0060059	BP	embryonic retina morphogenesis in camera-type eye
GO:0097529	BP	myeloid leukocyte migration
GO:0001894	BP	tissue homeostasis
GO:0071545	BP	inositol phosphate catabolic process
GO:0009612	BP	response to mechanical stimulus
GO:0019673	BP	GDP-mannose metabolic process
GO:1901264	BP	carbohydrate derivative transport
GO:0034637	BP	cellular carbohydrate biosynthetic process
GO:1900746	BP	regulation of vascular endothelial growth factor signaling pathway
GO:0009620	BP	response to fungus
GO:0043462	BP	regulation of ATP-dependent activity
GO:0046838	BP	phosphorylated carbohydrate dephosphorylation
GO:1903035	BP	negative regulation of response to wounding
GO:0001878	BP	response to yeast
GO:0048840	BP	otolith development
GO:1902547	BP	regulation of cellular response to vascular endothelial growth factor stimulus
GO:0043010	BP	camera-type eye development
GO:0030510	BP	regulation of BMP signaling pathway
GO:0010959	BP	regulation of metal ion transport
GO:0048706	BP	embryonic skeletal system development
GO:0000041	BP	transition metal ion transport

GO:0045216	BP	cell-cell junction organization
GO:0032963	BP	collagen metabolic process
GO:0006957	BP	complement activation, alternative pathway
GO:0005615	CC	extracellular space
GO:0031515	CC	tRNA (m1A) methyltransferase complex
GO:0043527	CC	tRNA methyltransferase complex
GO:0031012	CC	extracellular matrix
GO:0030312	CC	external encapsulating structure
GO:0005886	CC	plasma membrane
GO:0014069	CC	postsynaptic density
GO:0000159	CC	protein phosphatase type 2A complex
GO:0099572	CC	postsynaptic specialization
GO:0033017	CC	sarcoplasmic reticulum membrane
GO:0009986	CC	cell surface
GO:0016605	CC	PML body
GO:1903293	CC	phosphatase complex
GO:0008287	CC	protein serine/threonine phosphatase complex
GO:0005125	MF	cytokine activity
GO:0008009	MF	chemokine activity
GO:0042379	MF	chemokine receptor binding
GO:0001664	MF	G protein-coupled receptor binding
GO:0005126	MF	cytokine receptor binding

GO:0098772	MF	molecular function regulator activity
GO:0005457	MF	GDP-fucose transmembrane transporter activity
GO:0008934	MF	inositol monophosphate 1-phosphatase activity
GO:0001632	MF	leukotriene B4 receptor activity
GO:0004974	MF	leukotriene receptor activity
GO:0052834	MF	inositol monophosphate phosphatase activity
GO:0052832	MF	inositol monophosphate 3-phosphatase activity
GO:0052833	MF	inositol monophosphate 4-phosphatase activity
GO:0005153	MF	interleukin-8 receptor binding
GO:0016175	MF	superoxide-generating NAD(P)H oxidase activity
GO:0050480	MF	imidazolonepropionase activity
GO:0036080	MF	purine nucleotide-sugar transmembrane transporter activity
GO:0045236	MF	CXCR chemokine receptor binding
GO:0008446	MF	GDP-mannose 4,6-dehydratase activity
GO:0046872	MF	metal ion binding
GO:0043169	MF	cation binding
GO:0004896	MF	cytokine receptor activity
GO:0030546	MF	signaling receptor activator activity
GO:0048018	MF	receptor ligand activity
GO:0030545	MF	signaling receptor regulator activity
GO:0140375	MF	immune receptor activity
GO:0031994	MF	insulin-like growth factor I binding

GO:0031995	MF	insulin-like growth factor II binding
GO:0016494	MF	C-X-C chemokine receptor activity
GO:0002020	MF	protease binding
GO:0005338	MF	nucleotide-sugar transmembrane transporter activity
GO:0004222	MF	metalloendopeptidase activity
GO:0015020	MF	glucuronosyltransferase activity
GO:0042030	MF	ATPase inhibitor activity
GO:0030368	MF	interleukin-17 receptor activity
GO:0140678	MF	molecular function inhibitor activity
GO:0050664	MF	oxidoreductase activity, acting on NAD(P)H, oxygen as acceptor
GO:0005520	MF	insulin-like growth factor binding
GO:0005102	MF	signaling receptor binding
GO:0052745	MF	inositol phosphate phosphatase activity
GO:0016812	MF	hydrolase activity, acting on carbon-nitrogen (but not peptide) bonds, in cyclic amides
GO:0001637	MF	G protein-coupled chemoattractant receptor activity
GO:0004953	MF	icosanoid receptor activity
GO:0004950	MF	chemokine receptor activity
GO:0016805	MF	dipeptidase activity
GO:0019838	MF	growth factor binding
