# Supplementary material for: Transcriptome and 16S rRNA Amplicon Sequencing Analysis of Nutrition Metabolism in Silver Pomfret at Varying Flow Rates
Source: Animals (Basel). 2026 Jun 12;16(12):1818. doi: 10.3390/ani16121818 (PMC13295404; doi:10.3390/ani16121818)
Supplement: Supplementary file 1 [file animals-16-01818-s001.zip › Supplement Figure captions.pdf]

Figure S1 Length distribution and unigene and transcript distribution. D1_4/6/8L: liver for 400/600/800 L/h group at 4 weeks (first stage); D1_4/6 /8G: gut for 400/600/800 L/h group at 4 weeks (first stage); D1_4/6 /8M: muscle for 400/600/800 L/h group at 4 weeks (first stage); D2_4/6/8 L: liver for 400/600/800 L/h group at 8 weeks (second stage); D2_4/6 /8G: gut for 400/600/800 L/h group at 8 weeks (second stage); D2_4/6 /8M: muscle for 400/600/800 L/h group at 8 weeks (second stage).

Figure S2 Sample correlation heatmap, PCA, and inter group Venn plot between samples. D1_4/6/8L: liver for 400/600/800 L/h group at 4 weeks (first stage); D1_4/6 /8G: gut for 400/600/800 L/h group at 4 weeks (first stage); D1_4/6 /8M: muscle for 400/600/800 L/h group at 4 weeks (first stage); D2_4/6/8 L: liver for 400/600/800 L/h group at 8 weeks (second stage); D2_4/6 /8G: gut for 400/600/800 L/h group at 8 weeks (second stage); D2_4/6 /8M: muscle for 400/600/800 L/h group at 8 weeks (second stage).

Figure S3 DEG analysis among different groups. D1_4/6/8L: liver for 400/600/800 L/h group at 4 weeks (first stage); D1_4/6 /8G: gut for 400/600/800 L/h group at 4 weeks (first stage); D1_4/6 /8M: muscle for 400/600/800 L/h group at 4 weeks (first stage); D2_4/6/8 L: liver for 400/600/800 L/h group at 8 weeks (second stage); D2_4/6 /8G: gut for 400/600/800 L/h group at 8 weeks (second stage); D2_4/6 /8M: muscle for 400/600/800 L/h group at 8 weeks (second stage).

Figure S4 Network interaction and stability analysis at different flow rates. A/B: Network interaction; C/D/E/F: Network stability analysis, The robustness after removed random nodes./The absolute value of negative cohesion/AVD (Average Variance)/Vulnerability.

Figure S5 The neutral model analysis of the community assembly mechanism and quantitatively evaluates the ecological processes using iCAMP at different flow rates. C: The distribution of βNTI at different flow rates in different stages, where $\beta NTI < -2$ indicates homogeneous selection and $\beta NTI > 2$ indicates heterogeneous selection, $|\beta\ NTI| < 2$ indicates a neutral process. D: The proportion of community construction processes between utilization at different flow rates.
