# Supplementary material for: Transcriptome and 16S rRNA Amplicon Sequencing Analysis of Nutrition Metabolism in Silver Pomfret at Varying Flow Rates
Source: Animals (Basel). 2026 Jun 12;16(12):1818. doi: 10.3390/ani16121818 (PMC13295404; doi:10.3390/ani16121818)
Supplement: Supplementary file 1 [file animals-16-01818-s001.zip › Table S1.pdf]

Table S1 Quality analysis of transcriptome data in 54 libraries

Library	raw_reads	raw_bases	clean_reads	clean_bases	Error rate	Q20 (%)	Q30 (%)	MC content(%)
D1_4L1	44841024	6770994624	44161800	6571206518	0.0248	97.66	95.56	49.27
D1_4L2	47191496	7125915896	46146540	6841419058	0.0248	97.63	95.58	48.52
D1_4L3	50422554	7613805654	49448492	7390572028	0.0247	97.69	95.65	48.73
D1_4M1	44633804	6739704404	43533740	6501835520	0.0251	97.52	95.32	47.97
D1_4M2	50052210	7557883710	49074194	7325779551	0.0247	97.69	95.66	46.69
D1_4M3	46482678	7018884378	45659268	6811418468	0.0248	97.66	95.59	48.24
D1_4G1	52511726	7929270626	51508986	7682423592	0.0249	97.59	95.45	50.23
D1_4G2	54702262	8260041562	53625606	8015752892	0.025	97.56	95.39	50.08
D1_4G3	55375366	8361680266	54448206	8121452707	0.0248	97.65	95.57	49.16
D1_6L1	64839154	9790712254	63139508	9366463806	0.0246	97.72	95.75	49.53
D1_6L2	66739496	10077663896	65644808	9753550129	0.0246	97.74	95.75	49.98
D1_6L3	55152202	8327982502	53803862	7992503546	0.0246	97.71	95.73	48.78
D1_6M1	55214954	8337458054	53873196	7995972379	0.0246	97.74	95.79	44.58
D1_6M2	65593108	9904559308	63991814	9433119270	0.0244	97.82	95.88	49.48
D1_6M3	53605644	8094452244	52610348	7824822633	0.0246	97.75	95.78	43.58
D1_6G1	60551262	9143240562	59219864	8795560207	0.0249	97.6	95.47	50.08
D1_6G2	65135052	9835392852	63968102	9512178332	0.0246	97.71	95.71	50.7
D1_6G3	61722764	9320137364	60771010	9029094507	0.0248	97.66	95.58	50.07
D1_8L1	59368210	8964599710	58393264	8685577191	0.0246	97.74	95.78	48.43
D1_8L2	60312896	9107247296	59162668	8796957446	0.0245	97.79	95.85	49.78
D1_8L3	61139010	9231990510	59977722	8864362361	0.0247	97.69	95.67	48.4
D1_8M1	53354738	8056565438	52627920	7861551298	0.0246	97.75	95.75	49.04
D1_8M2	62364102	9416979402	61152742	9043372128	0.0248	97.68	95.62	49.16
D1_8M3	61684598	9314374298	60238090	8834172532	0.0246	97.74	95.78	47.83
D1_8G1	52809898	7974294598	51618878	7680102128	0.0248	97.65	95.56	50.65
D1_8G2	67850626	10245444526	66322872	9821651009	0.0248	97.66	95.61	50.15
D1_8G3	61720546	9319802446	60357738	8994066999	0.025	97.56	95.39	50.24
D2_4L1	54253286	8192246186	53548646	7990089683	0.0245	97.76	95.78	49.53
D2_4L2	63840410	9639901910	63053224	9410099264	0.0245	97.78	95.83	49.45
D2_4L3	53472816	8074395216	52653586	7868698679	0.0246	97.73	95.74	49.78
D2_4M1	51266260	7741205260	50225122	7484919372	0.0248	97.65	95.61	48.47
D2_4M2	60848746	9188160646	59805652	8908167124	0.0247	97.67	95.65	49.19
D2_4M3	59024874	8912755974	57847988	8613324318	0.0251	97.54	95.36	48.62
D2_4G1	51943716	7843501116	50643670	7533601651	0.0248	97.64	95.61	49.26
D2_4G2	53167686	8028320586	52199036	7768144698	0.0247	97.69	95.67	49.99
D2_4G3	52800722	7972909022	51947892	7699896680	0.0248	97.62	95.56	49.54
D2_6L1	68092218	10281924918	66394210	9861243427	0.0246	97.73	95.74	50.09
D2_6L2	62998180	9512725180	61844488	9202212228	0.0247	97.71	95.68	49.9
D2_6L3	59844920	9036582920	59006724	8784540716	0.0244	97.82	95.92	49.7
D2_6M1	61601428	9301815628	60759132	9033063735	0.0245	97.77	95.81	49.86
D2_6M2	62023758	9365587458	60819792	9045099395	0.0245	97.79	95.84	48.56

D2_6M3	50703230	7656187730	49831602	7406240151	0.0247	97.7	95.7	48.7
D2_6G1	71725238	10830510938	70427874	10461227735	0.0247	97.68	95.65	49.92
D2_6G2	55435336	8370735736	53807744	7984828842	0.025	97.53	95.41	48.94
D2_6G3	60081050	9072238550	59224604	8805791912	0.0249	97.61	95.48	50.2
D2_8L1	72808682	10994110982	72017578	10705913278	0.0242	97.9	96.09	49.64
D2_8L2	66914526	10104093426	65403166	9728915785	0.0247	97.67	95.62	49.43
D2_8L3	60515854	9137893954	59517592	8832425604	0.0246	97.74	95.76	49.98
D2_8M1	54049726	8161508626	53255686	7923435928	0.0247	97.67	95.62	48.93
D2_8M2	50297880	7594979880	49165770	7323127766	0.0249	97.59	95.48	48.61
D2_8M3	70297464	10614917064	69062240	10301952098	0.025	97.55	95.37	49.06
D2_8G1	59086342	8922037642	57722682	8576439115	0.0246	97.73	95.75	50.73
D2_8G2	53238408	8038999608	52318574	7784709091	0.0248	97.62	95.54	49.73
D2_8G3	56843042	8583299342	55558250	8252560467	0.0247	97.67	95.64	49.36
