# Supplementary material for: Transcriptome and 16S rRNA Amplicon Sequencing Analysis of Nutrition Metabolism in Silver Pomfret at Varying Flow Rates
Source: Animals (Basel). 2026 Jun 12;16(12):1818. doi: 10.3390/ani16121818 (PMC13295404; doi:10.3390/ani16121818)
Supplement: Supplementary file 1 [file animals-16-01818-s001.zip › Table S2.pdf]

Table S2 Top five GO terms in different comparing groups

ID	Description	P-value
**D1_6L vs. D1_4L**		
GO:0048500	signal recognition particle	0.00170697
GO:0005786	signal recognition particle, endoplasmic reticulum targeting	0.00170697
GO:0006613	cotranslational protein targeting to membrane	0.00170697
GO:0006612	protein targeting to membrane	0.00170697
GO:0006614	SRP-dependent cotranslational protein targeting to membrane	0.00170697
**D1_8L vs. D1_4L**		
GO:0008233	peptidase activity	1.34E-08
GO:0004175	endopeptidase activity	2.29E-08
GO:0004252	serine-type endopeptidase activity	4.33E-08
GO:0008236	serine-type peptidase activity	5.21E-08
GO:0017171	serine hydrolase activity	5.21E-08
**D1_8L vs. D1_6L**		
GO:0060046	regulation of acrosome reaction	0.001850007
GO:2000243	positive regulation of reproductive process	0.001850007
GO:2000344	positive regulation of acrosome reaction	0.001850007
GO:0035036	sperm-egg recognition	0.003696855
GO:0035803	egg coat formation	0.003696855
**D1_6G vs. D1_4G**		
GO:0005125	cytokine activity	0.000116149
GO:0007155	cell adhesion	0.000421414
GO:0008009	chemokine activity	0.000546174
GO:0042379	chemokine receptor binding	0.000546174
GO:0050900	leukocyte migration	0.002600186
**D1_8G vs. D1_4G**		
GO:0005634	nucleus	0.000168937
GO:0003700	DNA-binding transcription factor activity	0.000343633
GO:0003677	DNA binding	0.00071362
GO:0140110	transcription regulator activity	0.000726819
GO:0004879	nuclear receptor activity	0.001020271
**D1_8G vs. D1_6G**		
GO:0015453	oxidoreduction-driven active transmembrane transporter activity	5.08E-08
GO:0009055	electron transfer activity	2.12E-07
GO:0003954	NADH dehydrogenase activity	2.47E-07
GO:0050136	NADH dehydrogenase (quinone) activity	2.47E-07
GO:0003955	NAD(P)H dehydrogenase (quinone) activity	2.47E-07
**D1_6M vs. D1_4M**		
GO:0005581	collagen trimer	6.98E-06
GO:0005201	extracellular matrix structural constituent	9.58E-06
GO:0005861	troponin complex	0.000370706
GO:0001527	microfibril	0.000669624

GO:0031012	extracellular matrix	0.001124946
**D1_8M vs. D1_4M**		
GO:0140535	intracellular protein-containing complex	0.000193253
GO:0034660	ncRNA metabolic process	7.11E-05
GO:0006399	tRNA metabolic process	0.000136471
GO:0030314	junctional membrane complex	0.000810577
GO:0032045	guanyl-nucleotide exchange factor complex	0.001936483
**D1_8M vs. D1_6M**		
GO:0009311	oligosaccharide metabolic process	0.001171521
GO:0018394	peptidyl-lysine acetylation	0.00343485
GO:0006475	internal protein amino acid acetylation	0.00343485
GO:0016573	histone acetylation	0.00343485
GO:0018393	internal peptidyl-lysine acetylation	0.00343485
**D2_6L vs. D2_4L**		
GO:0030574	collagen catabolic process	0.003696855
GO:0006616	SRP-dependent cotranslational protein targeting to membrane, translocation	0.009218488
GO:0061371	determination of heart left/right asymmetry	0.009218488
GO:0032963	collagen metabolic process	0.011052746
GO:1903706	regulation of hemopoiesis	0.014711862
**D2_8L vs. D2_4L**		
GO:0070571	negative regulation of neuron projection regeneration	0.000853849
GO:0046677	response to antibiotic	0.000853849
GO:0048681	negative regulation of axon regeneration	0.000853849
GO:0048679	regulation of axon regeneration	0.000853849
GO:0070570	regulation of neuron projection regeneration	0.000853849
**D2_8L vs. D2_6L**		
GO:0006564	L-serine biosynthetic process	0.001991465
GO:0006563	L-serine metabolic process	0.004972288
GO:0042157	lipoprotein metabolic process	0.00695526
GO:0009070	serine family amino acid biosynthetic process	0.007945476
GO:0006123	mitochondrial electron transport, cytochrome c to oxygen	0.009923368
**D2_6G vs. D2_4G**		
GO:0042151	nematocyst	0.003411996
GO:0044217	other organism part	0.003411996
GO:0044218	other organism cell membrane	0.003411996
GO:0051715	cytolysis in another organism	0.003411996
GO:0031640	killing of cells of another organism	0.003411996
**D2_8G vs. D2_4G**		
GO:0005506	iron ion binding	2.76E-06
GO:0016491	oxidoreductase activity	1.49E-05
GO:0042887	amide transmembrane transporter activity	0.000283469
GO:0016021	integral component of membrane	0.000477476
GO:0031224	intrinsic component of membrane	0.000495324

**D2_8G vs. D2_6G**		
GO:0005506	iron ion binding	0.000174955
GO:0016176	superoxide-generating NADPH oxidase activator activity	0.00020052
GO:0008610	lipid biosynthetic process	5.15E-05
GO:0046914	transition metal ion binding	0.000656388
GO:0016491	oxidoreductase activity	0.000839217
**D2_6M vs. D2_4M**		
GO:2001295	malonyl-CoA biosynthetic process	0.002846165
GO:0070973	protein localization to endoplasmic reticulum exit site	0.002846165
GO:2001293	malonyl-CoA metabolic process	0.002846165
GO:0070647	protein modification by small protein conjugation or removal	0.003781423
GO:0044260	cellular macromolecule metabolic process	0.012377852
**D2_8M vs. D2_4M**		
GO:0051641	cellular localization	0.000531173
GO:0071705	nitrogen compound transport	0.000657727
GO:0015031	protein transport	0.001020747
GO:0045184	establishment of protein localization	0.001101706
GO:0071702	organic substance transport	0.001385107
**D2_8M vs. D2_6M**		
GO:0006222	UMP biosynthetic process	1.89E-05
GO:0009173	pyrimidine ribonucleoside monophosphate metabolic process	1.89E-05
GO:0009130	pyrimidine nucleoside monophosphate biosynthetic process	1.89E-05
GO:0009129	pyrimidine nucleoside monophosphate metabolic process	1.89E-05
GO:0046049	UMP metabolic process	1.89E-05
