# Supplementary material for: Transcriptome and 16S rRNA Amplicon Sequencing Analysis of Nutrition Metabolism in Silver Pomfret at Varying Flow Rates
Source: Animals (Basel). 2026 Jun 12;16(12):1818. doi: 10.3390/ani16121818 (PMC13295404; doi:10.3390/ani16121818)
Supplement: Supplementary file 1 [file animals-16-01818-s001.zip › Table S3.pdf]

**Table S3 Top five KEGG pathways in different comparing groups**

| ID | KEGG pathway | P-value |
|---|---|---|
| **D1_6L vs. D1_4L** | | |
| map04141 | Protein processing in endoplasmic reticulum | 0.001445975 |
| map00051 | Fructose and mannose metabolism | 0.064118681 |
| map01250 | Biosynthesis of nucleotide sugars | 0.045941827 |
| map00520 | Amino sugar and nucleotide sugar metabolism | 0.056887253 |
| map04612 | Antigen processing and presentation | 0.10297122 |
| **D1_8L vs. D1_4L** | | |
| map04974 | Protein digestion and absorption | 1.35E-15 |
| map04972 | Pancreatic secretion | 6.87E-14 |
| map05164 | Influenza A | 0.000188067 |
| map04080 | Neuroactive ligand-receptor interaction | 0.00142533 |
| map04610 | Complement and coagulation cascades | 0.033480952 |
| **D1_8L vs. D1_6L** | | |
| map00260 | Glycine, serine and threonine metabolism | 0.003579311 |
| map00760 | Nicotinate and nicotinamide metabolism | 0.083302295 |
| map01523 | Antifolate resistance | 0.065385128 |
| map00410 | beta-Alanine metabolism | 0.079490782 |
| map04216 | Ferroptosis | 0.103376754 |
| **D1_6G vs. D1_4G** | | |
| map04060 | Cytokine-cytokine receptor interaction | 4.28E-05 |
| map04657 | IL-17 signaling pathway | 0.000123553 |
| map05417 | Lipid and atherosclerosis | 0.000212771 |
| map05144 | Malaria | 0.000946974 |
| map04964 | Proximal tubule bicarbonate reclamation | 0.006286175 |
| **D1_8G vs. D1_4G** | | |
| map04010 | MAPK signaling pathway | 0.000228321 |
| map04974 | Protein digestion and absorption | 0.000830814 |
| map04972 | Pancreatic secretion | 0.000752873 |
| map04657 | IL-17 signaling pathway | 0.002083401 |
| map04380 | Osteoclast differentiation | 0.00518882 |
| **D1_8G vs. D1_6G** | | |
| map00190 | Oxidative phosphorylation | 9.74E-21 |
| map05415 | Diabetic cardiomyopathy | 1.14E-16 |
| map05208 | Chemical carcinogenesis - reactive oxygen species | 2.56E-16 |
| map05012 | Parkinson disease | 1.55E-15 |
| map04714 | Thermogenesis | 1.35E-15 |
| **D1_6M vs. D1_4M** | | |
| map04512 | ECM-receptor interaction | 4.27E-09 |
| map04974 | Protein digestion and absorption | 9.97E-09 |
| map04510 | Focal adhesion | 2.77E-06 |
| map04261 | Adrenergic signaling in cardiomyocytes | 6.28E-06 |

| | | |
|---|---|---|
| map05165 | Human papillomavirus infection | 0.000125138 |
| **D1_8M vs. D1_4M** | | |
| map04974 | Protein digestion and absorption | 8.93E-05 |
| map04144 | Endocytosis | 0.001300577 |
| map00190 | Oxidative phosphorylation | 0.000690474 |
| map04721 | Synaptic vesicle cycle | 0.001046272 |
| map00520 | Amino sugar and nucleotide sugar metabolism | 0.003277267 |
| **D1_8M vs. D1_6M** | | |
| map00760 | Nicotinate and nicotinamide metabolism | 0.006495217 |
| map05168 | Herpes simplex virus 1 infection | 0.999964368 |
| map00520 | Amino sugar and nucleotide sugar metabolism | 0.005438595 |
| map04972 | Pancreatic secretion | 0.015223157 |
| map00510 | N-Glycan biosynthesis | 0.017243069 |
| **D2_6L vs. D2_4L** | | |
| map05322 | Systemic lupus erythematosus | 0.002572937 |
| map05133 | Pertussis | 0.002413967 |
| map05150 | Staphylococcus aureus infection | 0.002020418 |
| map04610 | Complement and coagulation cascades | 0.006985719 |
| map05142 | Chagas disease | 0.006216646 |
| **D2_8L vs. D2_4L** | | |
| map05215 | Prostate cancer | 0.005781135 |
| map04657 | IL-17 signaling pathway | 0.003488544 |
| map04668 | TNF signaling pathway | 0.00519321 |
| map04915 | Estrogen signaling pathway | 0.008875747 |
| map05417 | Lipid and atherosclerosis | 0.0015055 |
| **D2_8L vs. D2_6L** | | |
| map00750 | Vitamin B6 metabolism | 0.00501941 |
| map04145 | Phagosome | 0.157032226 |
| map00190 | Oxidative phosphorylation | 0.153337593 |
| map05152 | Tuberculosis | 0.150247399 |
| map00010 | Glycolysis / Gluconeogenesis | 0.095451493 |
| **D2_6G vs. D2_4G** | | |
| map05414 | Dilated cardiomyopathy | 0.142164447 |
| map05225 | Hepatocellular carcinoma | 0.140914679 |
| map04068 | FoxO signaling pathway | 0.138410135 |
| map04926 | Relaxin signaling pathway | 0.131488086 |
| map04066 | HIF-1 signaling pathway | 0.135898899 |
| **D2_8G vs. D2_4G** | | |
| map00100 | Steroid biosynthesis | 1.41E-07 |
| map04216 | Ferroptosis | 8.49E-06 |
| map00590 | Arachidonic acid metabolism | 4.13E-05 |
| map05208 | Chemical carcinogenesis - reactive oxygen species | 3.44E-05 |
| map00982 | Drug metabolism - cytochrome P450 | 5.27E-05 |
| **D2_8G vs. D2_6G** | | |

| | | |
|---|---|---|
| map00100 | Steroid biosynthesis | 1.62E-13 |
| map00260 | Glycine, serine and threonine metabolism | 0.000392126 |
| map05417 | Lipid and atherosclerosis | 0.000638005 |
| map04613 | Neutrophil extracellular trap formation | 0.001546322 |
| map05140 | Leishmaniasis | 0.002252589 |
| **D2_6M vs. D2_4M** | | |
| map05144 | Malaria | 0.144574634 |
| map00380 | Tryptophan metabolism | 0.152772476 |
| map00760 | Nicotinate and nicotinamide metabolism | 0.140446762 |
| map00790 | Folate biosynthesis | 0.095914515 |
| map04391 | Hippo signaling pathway - fly | 0.186766938 |
| **D2_8M vs. D2_4M** | | |
| map04141 | Protein processing in endoplasmic reticulum | 1.52E-06 |
| map05169 | Epstein-Barr virus infection | 0.000610878 |
| map03050 | Proteasome | 0.001001925 |
| map03060 | Protein export | 0.004173576 |
| map05160 | Hepatitis C | 0.008907695 |
| **D2_8M vs. D2_6M** | | |
| map04141 | Protein processing in endoplasmic reticulum | 5.52E-12 |
| map05417 | Lipid and atherosclerosis | 1.16E-05 |
| map03008 | Ribosome biogenesis in eukaryotes | 2.59E-05 |
| map04010 | MAPK signaling pathway | 9.68E-05 |
| map05219 | Bladder cancer | 0.000174095 |
