# Supplementary material for: Transcriptome and 16S rRNA Amplicon Sequencing Analysis of Nutrition Metabolism in Silver Pomfret at Varying Flow Rates
Source: Animals (Basel). 2026 Jun 12;16(12):1818. doi: 10.3390/ani16121818 (PMC13295404; doi:10.3390/ani16121818)
Supplement: Supplementary file 1 [file animals-16-01818-s001.zip › Table S4.pdf]

Table S4 The α diversity of silver pomfret at different flow rates

Sample ID	richness	chao1	ACE	shannon	simpson
D1_4_1	1107	1227.86	1220.41	6.269414	0.996469
D1_4_2	1165	1236.02	1221.95	6.424333	0.996703
D1_4_3	1031	1123.18	1125.76	6.165976	0.996066
D1_4_4	1174	1229.04	1229.44	6.617754	0.998036
D1_4_5	1201	1320.64	1307.28	6.424413	0.996938
D1_6_1	899	1167.2	1208.93	5.409528	0.989608
D1_6_2	1172	1238.85	1232.55	6.385763	0.996374
D1_6_3	614	1058.42	1134.22	4.922772	0.986137
D1_6_4	1151	1273.48	1245.86	6.514613	0.997741
D1_6_5	241	250	243.673	4.743835	0.985391
D1_8_1	1267	1431.23	1420.23	6.420467	0.996876
D1_8_2	739	971.2	980.756	5.659206	0.994111
D1_8_3	1335	1506.11	1534.65	6.458186	0.997111
D1_8_4	1459	1547.55	1536.37	6.856805	0.998366
D1_8_5	918	1248.17	1312.85	5.835395	0.994773
D2_4_1	1492	1770.93	1745.45	6.773195	0.998306
D2_4_2	532	754.230	667.947	5.782028	0.995978
D2_4_3	1302	1731.76	1679.2	6.5216	0.997656
D2_4_4	1657	1855.06	1820.36	7.024649	0.99878
D2_4_5	1177	1276.22	1246.86	6.573558	0.997787
D2_6_1	1235	1438.72	1429.28	6.389416	0.99694
D2_6_2	1442	1647.9	1667.25	6.65622	0.997879
D2_6_3	1419	1541.04	1532.71	6.798685	0.998348
D2_6_4	933	1124.07	1156.80	5.94294	0.995536
D2_6_5	1510	1784.00	1786.33	6.181913	0.985788
D2_8_1	1008	1133.11	1124.95	6.089265	0.995545
D2_8_2	1204	1324.94	1320.58	6.371883	0.996083

D2_8_3	1127	1310.80	1326.41	6.087345	0.995558
D2_8_4	1409	1615.89	1626.51	6.600298	0.997468
D2_8_5	586	872.263	715.454	5.338251	0.99075
