# Supplementary figures and images for: Transcriptome and 16S rRNA Amplicon Sequencing Analysis of Nutrition Metabolism in Silver Pomfret at Varying Flow Rates
Source: Animals (Basel). 2026 Jun 12;16(12):1818. doi: 10.3390/ani16121818 (PMC13295404; doi:10.3390/ani16121818)

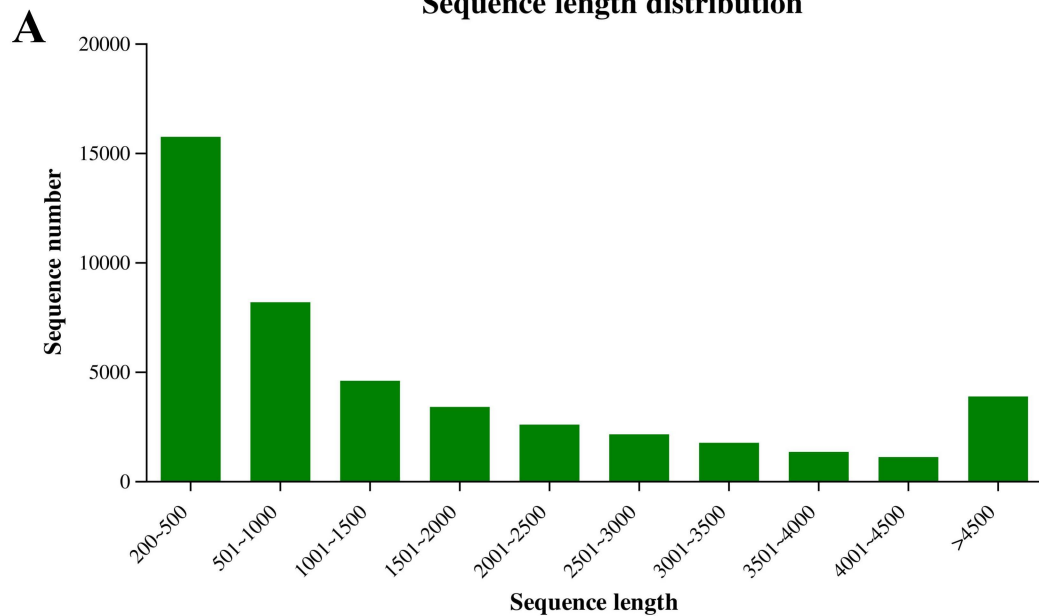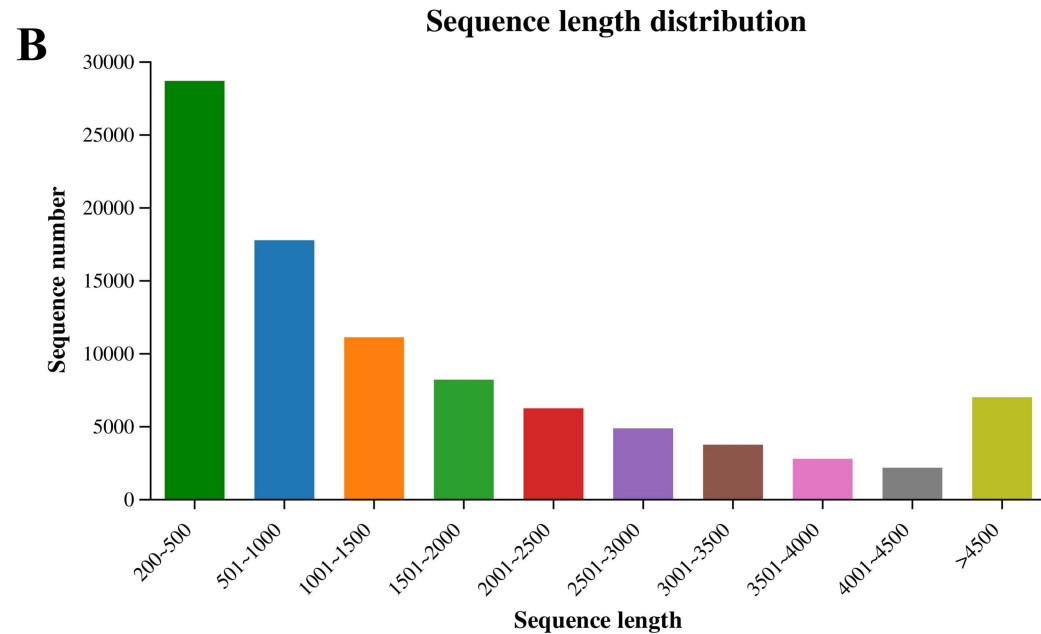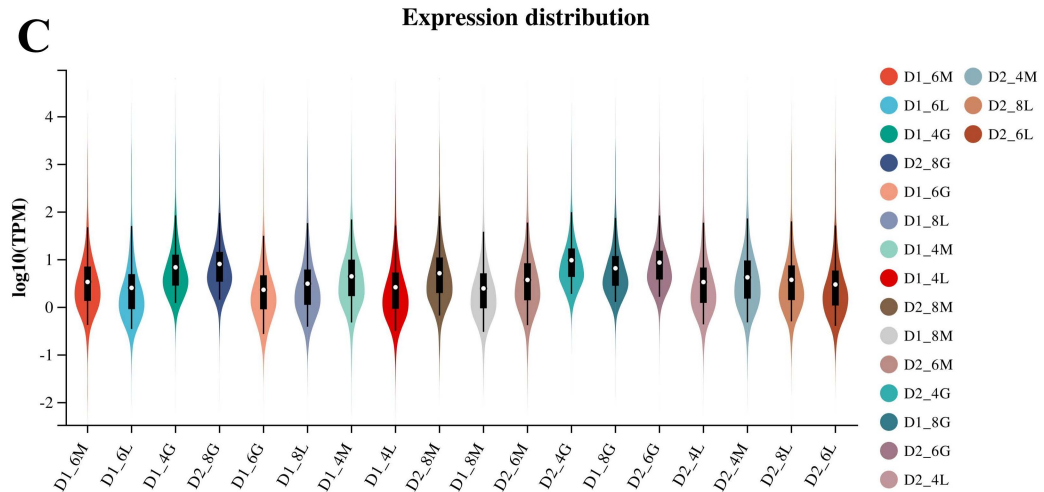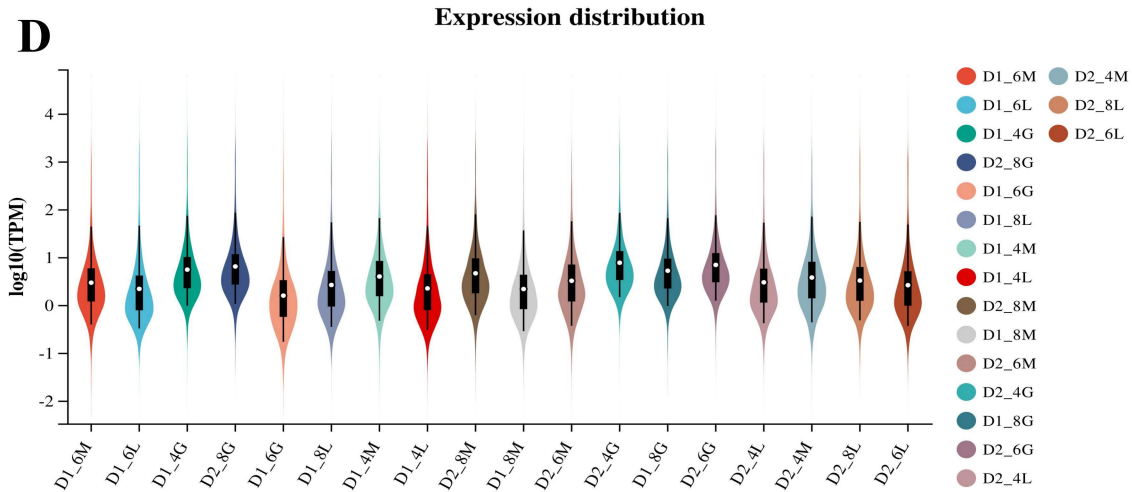

Supplement: Supplementary file 1 [file animals-16-01818-s001.zip › Figure S1.pdf]

**A****Differential statistics**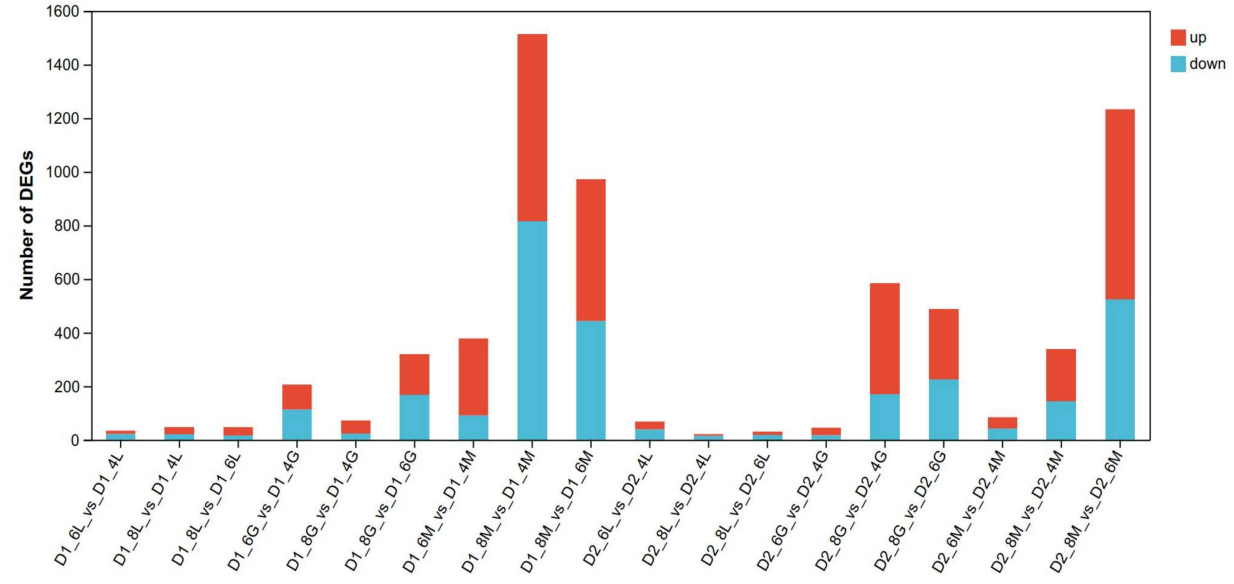**B**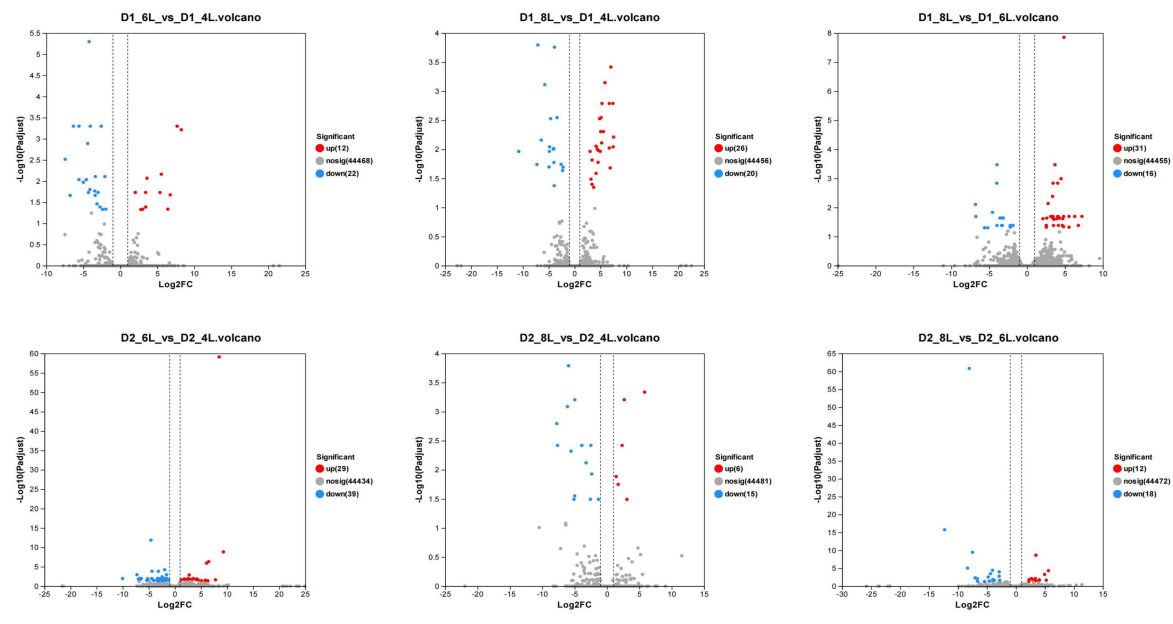**C**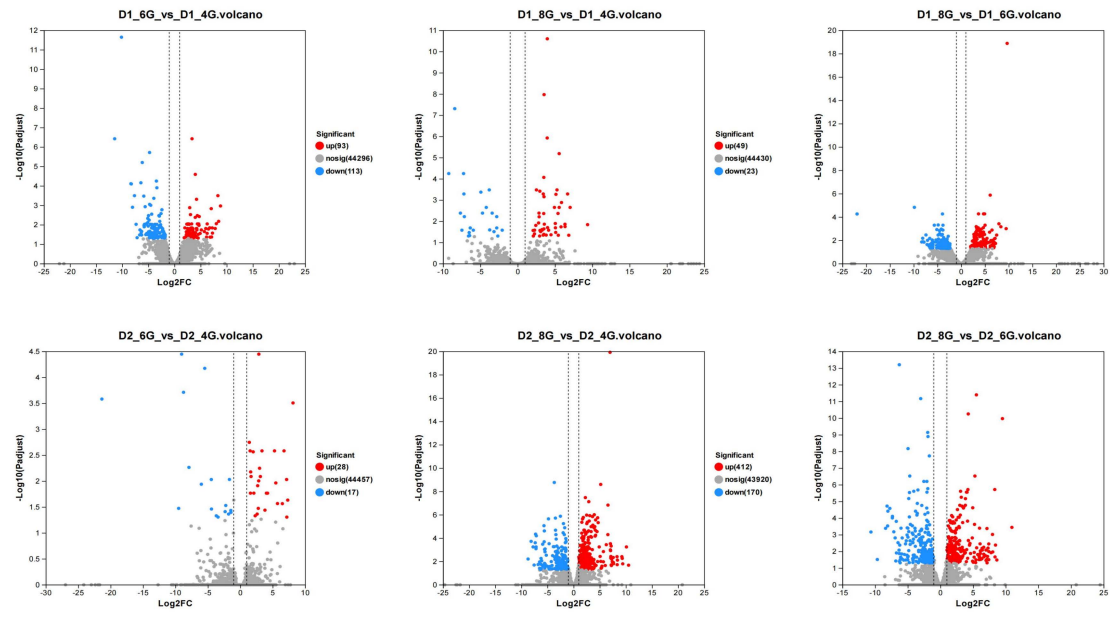**D**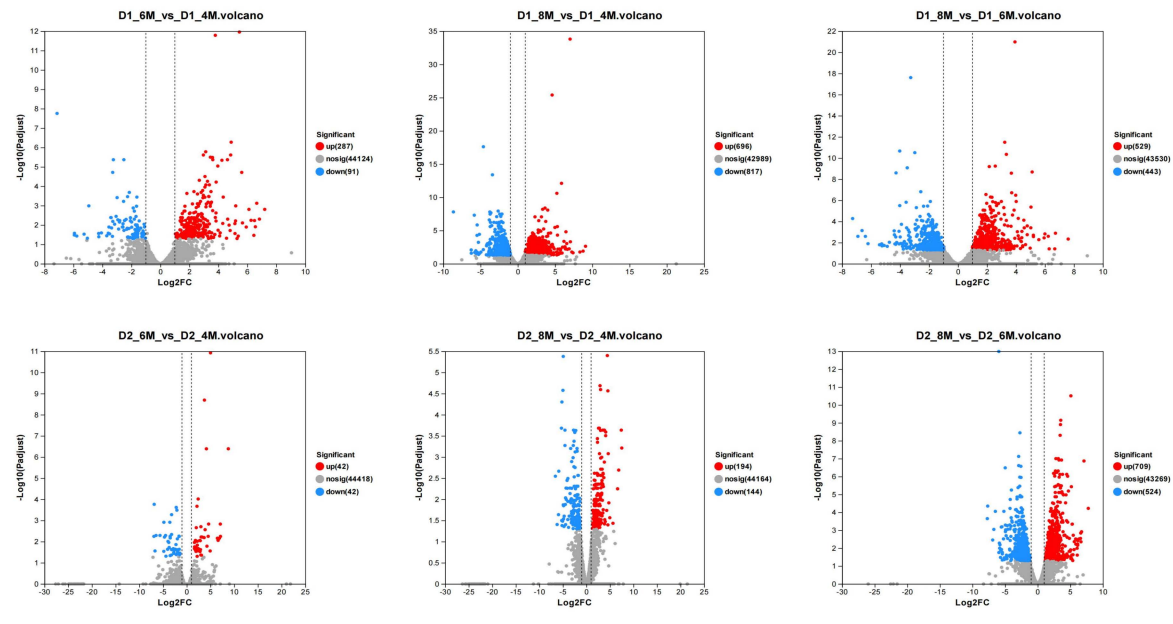

Supplement: Supplementary file 1 [file animals-16-01818-s001.zip › Figure S3.pdf]

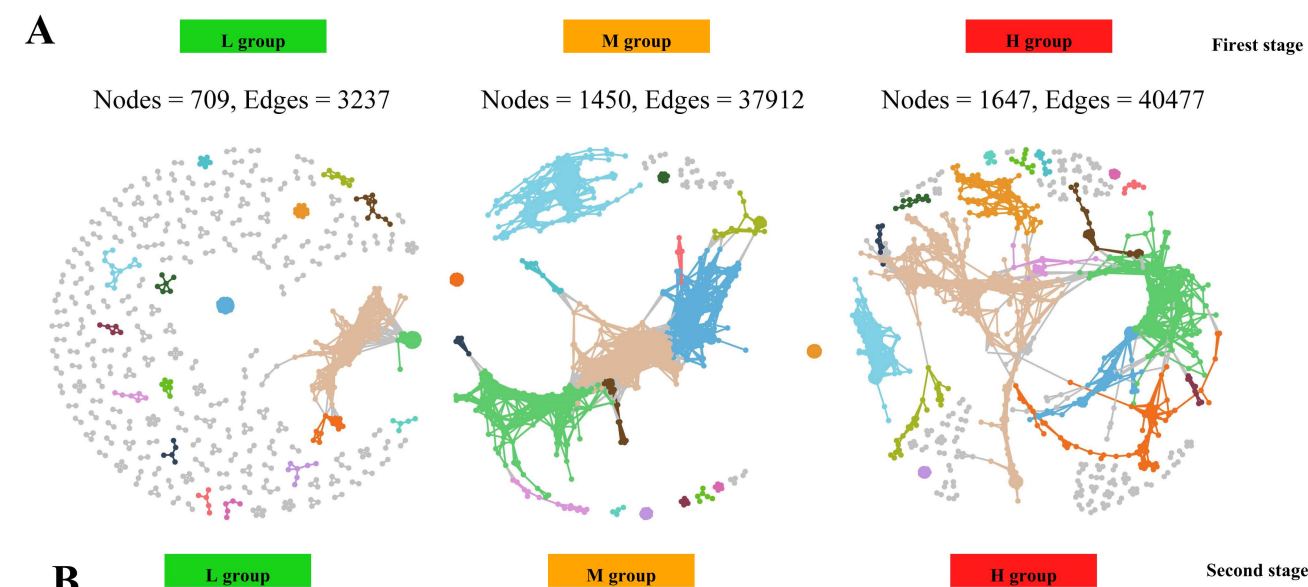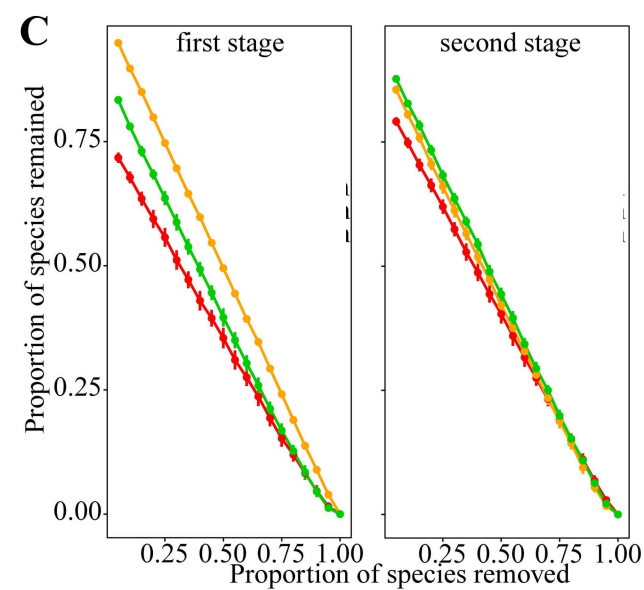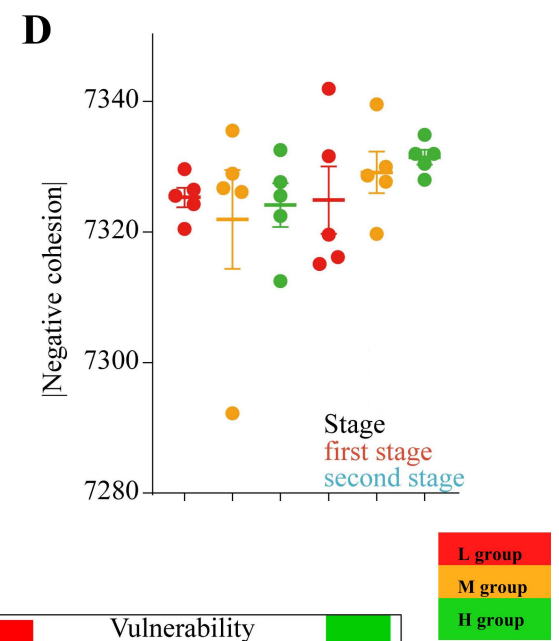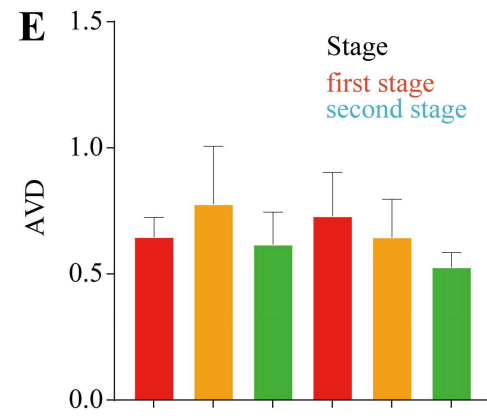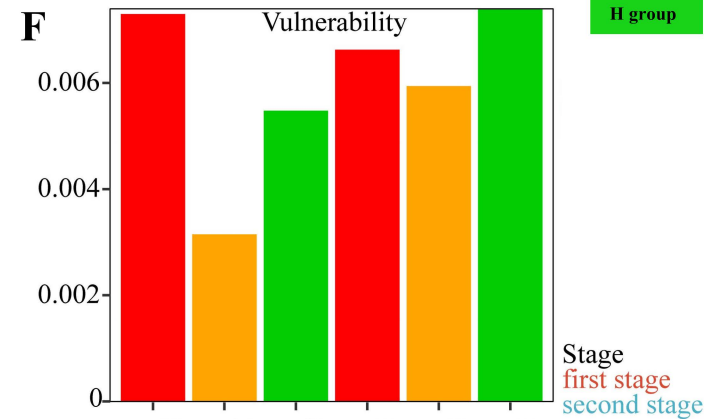

Supplement: Supplementary file 1 [file animals-16-01818-s001.zip › Figure S4.pdf]

**A**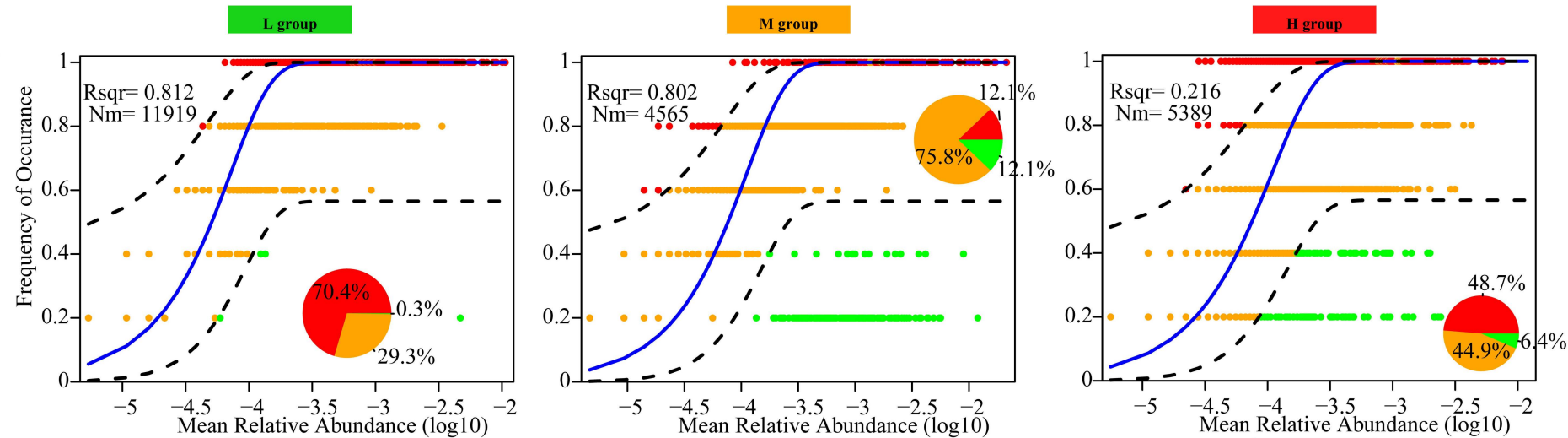**B**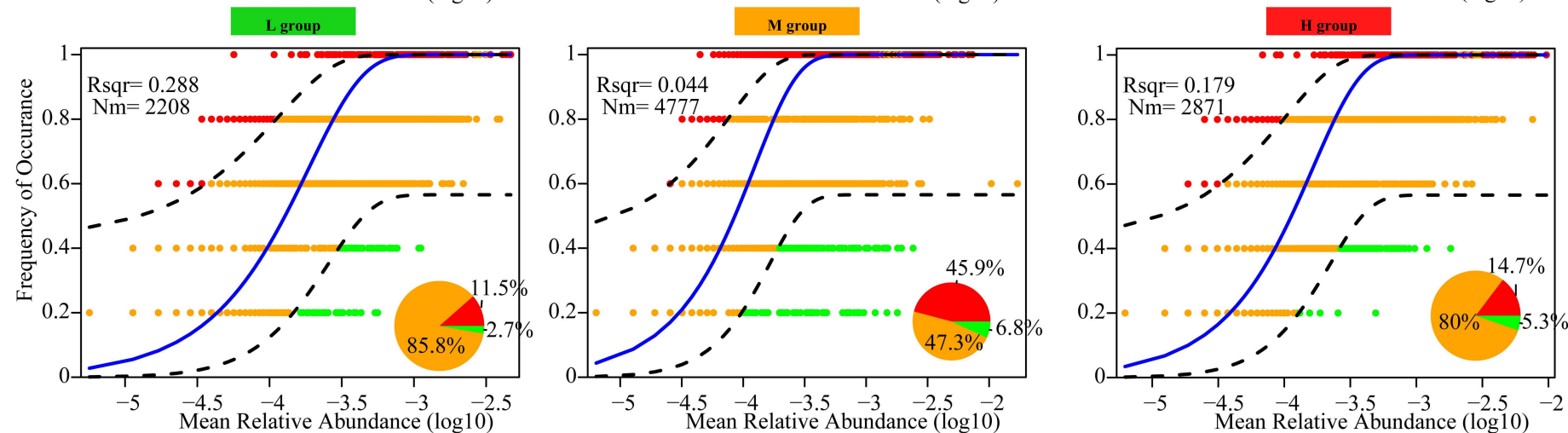**C**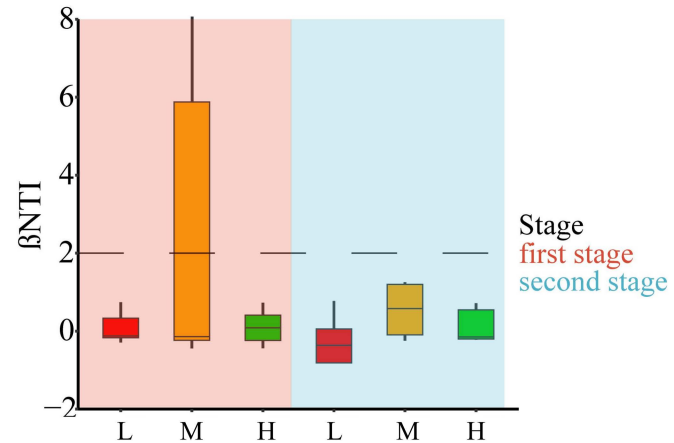**D**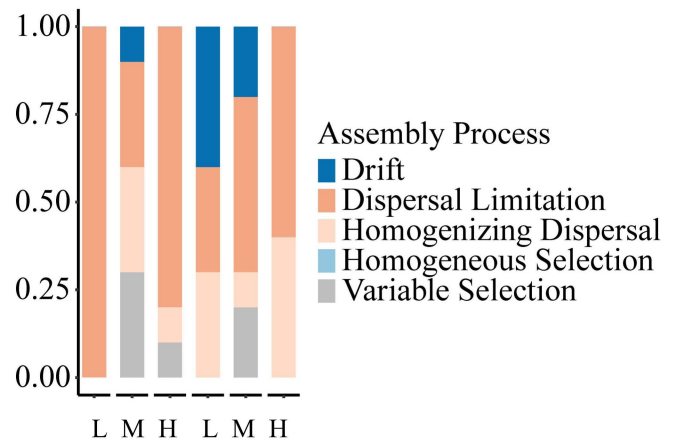

Supplement: Supplementary file 1 [file animals-16-01818-s001.zip › Figure S5.pdf]
