# Supplementary material for: Transcriptome and 16S rRNA Amplicon Sequencing Analysis of Nutrition Metabolism in Silver Pomfret at Varying Flow Rates
Source: Animals (Basel). 2026 Jun 12;16(12):1818. doi: 10.3390/ani16121818 (PMC13295404; doi:10.3390/ani16121818)
Supplement: Supplementary file 1 [file Figure S2.pdf › Figure S2.pdf]

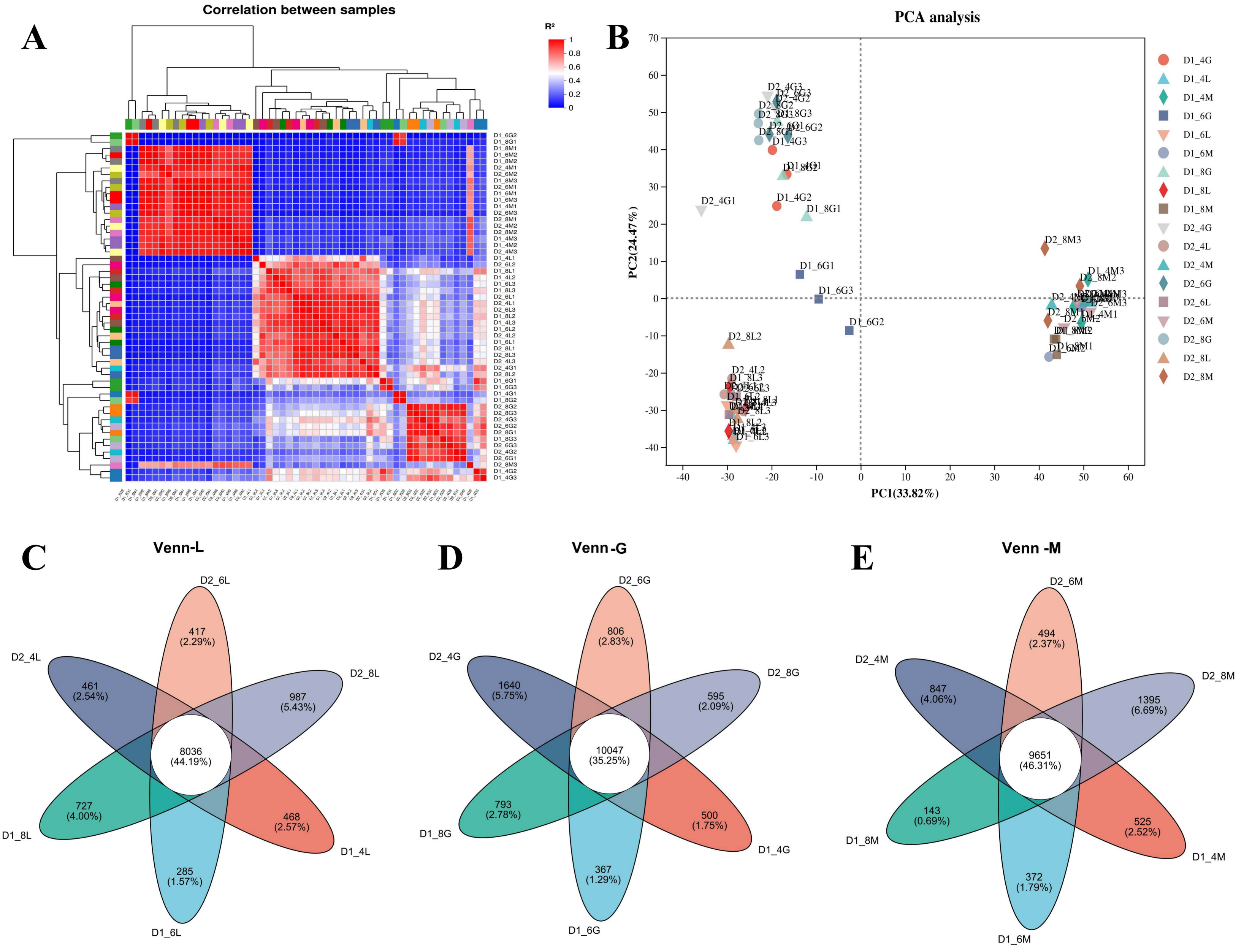

A
Correlation between samples
R²
1
0.8
0.6
0.4
0.2
0
B
PCA analysis
PC1(33.82%)
PC2(24.47%)
D1_4G
D1_4L
D1_4M
D1_6G
D1_6L
D1_6M
D1_8G
D1_8L
D1_8M
D2_4G
D2_4L
D2_4M
D2_6G
D2_6L
D2_6M
D2_8G
D2_8L
D2_8M
C
Venn-L
D2_6L
D2_4L
D2_8L
D1_8L
D1_4L
D1_6L
417 (2.29%)
461 (2.54%)
987 (5.43%)
8036 (44.19%)
727 (4.00%)
468 (2.57%)
285 (1.57%)
D
Venn-G
D2_6G
D2_4G
D2_8G
D1_8G
D1_4G
D1_6G
806 (2.83%)
1640 (5.75%)
595 (2.09%)
10047 (35.25%)
793 (2.78%)
500 (1.75%)
367 (1.29%)
E
Venn -M
D2_6M
D2_4M
D2_8M
D1_8M
D1_4M
D1_6M
494 (2.37%)
847 (4.06%)
1395 (6.69%)
9651 (46.31%)
143 (0.69%)
525 (2.52%)
372 (1.79%)
